# SOAPnuke: a MapReduce acceleration-supported software for integrated quality control and preprocessing of high-throughput sequencing data

**DOI:** 10.1093/gigascience/gix120

**Published:** 2017-12-04

**Authors:** Yuxin Chen, Yongsheng Chen, Chunmei Shi, Zhibo Huang, Yong Zhang, Shengkang Li, Yan Li, Jia Ye, Chang Yu, Zhuo Li, Xiuqing Zhang, Jian Wang, Huanming Yang, Lin Fang, Qiang Chen

**Affiliations:** 1 BGI-Shenzhen, Shenzhen 518083; 2 Geneplus-Beijing, Beijing 102206; 3 Department of Oncology, Fujian Medical University Union Hospital, Fuzhou 350001; 4 Fujian Key Laboratory of Translational Cancer Medicine, Fuzhou 350014; 5 Department of Stem Cell Research Institute, Fujian Medical University Stem Cell Research Institute, Fuzhou 350000; 6 Collaborative Innovation Center of High Performance Computing, National University of Defense Technology, Changsha 410073; 7 Intel China Ltd., Shanghai 200336; 8 Guangdong Provincial Hospital of Chinese Medicine, Guangzhou 510120; 9 Department of Surgery, Faculty of Medicine, The Chinese University of Hong Kong, Hong Kong; 10 James D. Watson Institute of Genome Sciences, Hangzhou 310058, China

**Keywords:** high-throughput sequencing, quality control, preprocessing, MapReduce

## Abstract

Quality control (QC) and preprocessing are essential steps for sequencing data analysis to ensure the accuracy of results. However, existing tools cannot provide a satisfying solution with integrated comprehensive functions, proper architectures, and highly scalable acceleration. In this article, we demonstrate SOAPnuke as a tool with abundant functions for a “QC-Preprocess-QC” workflow and MapReduce acceleration framework. Four modules with different preprocessing functions are designed for processing datasets from genomic, small RNA, Digital Gene Expression, and metagenomic experiments, respectively. As a workflow-like tool, SOAPnuke centralizes processing functions into 1 executable and predefines their order to avoid the necessity of reformatting different files when switching tools. Furthermore, the MapReduce framework enables large scalability to distribute all the processing works to an entire compute cluster.

We conducted a benchmarking where SOAPnuke and other tools are used to preprocess a ∼30× NA12878 dataset published by GIAB. The standalone operation of SOAPnuke struck a balance between resource occupancy and performance. When accelerated on 16 working nodes with MapReduce, SOAPnuke achieved ∼5.7 times the fastest speed of other tools.

## Background

High-throughput sequencing (HTS) instruments have enabled many large-scale studies and generated enormous amounts of data [1–3]. However, the presence of low-quality bases, sequence artifacts, and sequence contamination can introduce serious negative impact on downstream analyses. Thus, QC and preprocessing of raw data serve as the critical steps to initiate analysis pipelines [4, 5]. QC investigates several statistics of datasets to ensure data quality, and preprocessing trims off undesirable terminal fragments and filters out substandard reads [6]. We have conducted a survey on 31 existing tools, and widely shared functions are listed in Supplementary Material 1.

Existing tools for QC and preprocessing can be divided into 2 categories according to their structures: toolkit and workflow. Toolkit-like software provides multiple executables such as statistics computer, clipper, and filtrator [7–15]. In practice, raw data are processed by a few individual executables in sequence. Comparatively, workflow-like software offers an integral workflow where functions are performed in predefined order [6, 16–37].

However, both categories have their own demerits. When using toolkit-like software, it is complex and error-prone to write additional scripts to wrap executables. Moreover, it consumes much time to generate and read intermediate files, which is hard for acceleration. Besides, the same variables could possibly be computed repetitively. For instance, the average quality score of each read is necessary for counting quality score distribution by reads and filtering reads based on average quality scores. It has to be counted twice if these 2 functions are implemented by different toolkits.

For workflow-like tools, an optimal architecture is required because the orders of functions are fixed. Most of the existing tools successively perform QC and preprocessing without complete statistics of preprocessed datasets. If the preprocessing operation is not suitable for a given dataset, the problem can only be revealed by downstream analyses.

Datasets sequenced from various samples may require different processing functions or parameters. Existing workflow-like tools mostly support genomics data processing; only a few of them are developed for other types of studies, such as RNA-seq and metagenomics data. For example, RObiNA [22] provides 4 preprocessing modules to combined for different RNA-Seq Data. PrinSeq [6] offers a QC stat, dinucleotide odds ratios, to show how the dataset might be related to other viral/microbial metagenomes. However, there is still no single tool supporting multiple data types.

Several tools have made certain progress in overcoming the limitations mentioned above. Galaxy [37] is a web-based platform incorporating various existing toolkit-like softwares. Users can conveniently concatenate tools into a pipeline on the web interface. NGS QC toolkit [16] offers a workflow with QC on both raw and preprocessed datasets, though there are few preprocessing functions.

In terms of software acceleration, only multithreading is adopted by existing tools [14–16, 24–28]. This approach only works for standalone operation and is limited by the maximum number of processors in 1 computer server. It may be incompetent when dealing with the huge present and potential volume of sequencing datasets.

To solve these problems, we have developed a workflow-like tool, SOAPnuke, for integrated QC and preprocessing of large HTS datasets. Similar to NGS QC toolkit, SOAPnuke performs 2-step QC. Trimming, filtering, and other frequently used functions are integrated in our program. Four modules are designed to handle genomic, metagenomic, DGE, and sRNA datasets, respectively. In addition, SOAPnuke is extended to multiple working nodes for parallel computing using Hadoop MapReduce framework.

## Methods

### QC and preprocessing

**Figure 1:**
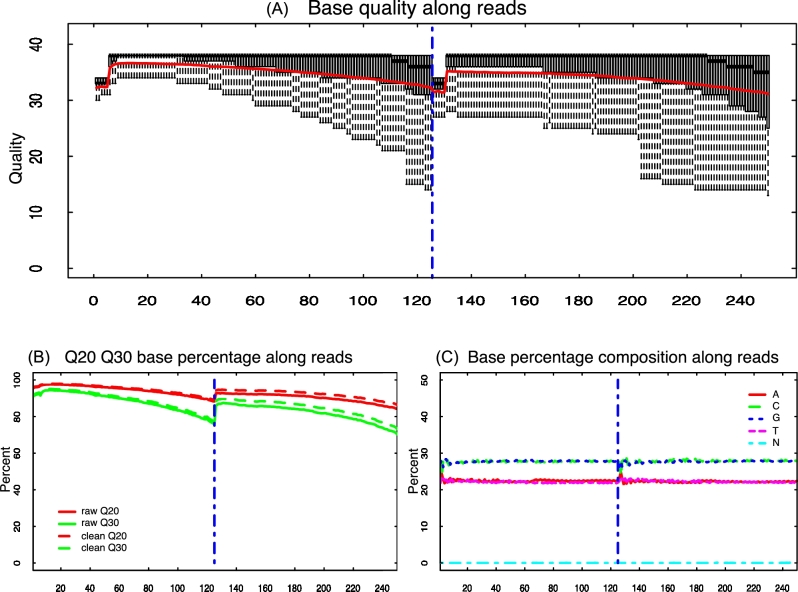
An example of QC complex statistics. (**A**) Per-base quality distribution of raw paired-end reads. (**B**) Per-base Q20 and Q30 of raw and preprocessed paired-end reads. (**C**) Per-base base composition distribution of raw paired-end reads.

SOAPnuke (SOAPnuke, RRID:SCR_015025) was developed to summarize statistics of both raw and preprocessed data. Basic statistics are comprised of the number of sequences and bases, base composition, Q20 and Q30, and filtering information. Complex statistics include the distribution of quality score and base composition distribution for each position. For the quality score distribution, Q20 and Q30 for each position are plotted in a line chart, and the quantiles of the quality are represented in a boxplot. And for the base composition distribution, an overlapping histogram is used to display base composition distribution for each position. These calculations are conducted by C++, and the plots are generated by R 3.3.2 [38]. An example of the 2 plots is shown in Fig. 1. A comprehensive list of statistics available in SOAPnuke is included in Additional file 2. Statistics of preprocessed data are compared with some preset thresholds. A warning message will be issued if the median score of any position in per-base quality distribution is lower than 25, and a failure will be issued if it is lower than 20. For per-base base composition, a warning will be raised if the difference between A and T, or G and C, in any position is greater than 10%, or a failure will be issued if it is greater than 20%.

In the step of preprocessing, those undesirable terminal fragments are trimmed off, substandard reads are filtered out, and certain transform operations are applied. On both ends of reads, bases of assigned number or of quality lower than the threshold will be trimmed off. Sequencing adapters can be aligned, where mismatch is supported while no INDEL is tolerated, and cut to the 3’ end. Filtering can be performed on reads with adapter, short length, too many ambiguous bases, low–average quality, or too many low-quality bases. The sequencing batches, such as tile of Illumina sequencer [39] and fov (field of view) of BGI sequencer [40], with unfavorable sequencing quality can be assigned so that the corresponding sequences will be discarded. In addition, reads with identical nucleotides can be deduplicated to keep only 1 copy. Transformation comprises quality system conversion, interconversion between DNA and RNA, and compression of output with gzip, etc. Additional file 3 lists the above preprocessing functions and their parameters.

### Module design

To improve processing performance of different types of data, 4 modules are specialized in SOAPnuke, including the General, DGE, sRNA, and Meta modules. (1) The General module can handle most of the DNA re-sequencing datasets, as described in the section of QC & PROCESSING.

(2) DGE profiling generates a single-end read that has a “CATG” segment neighboring the targeted sequences of 17 base pairs [41]. By default, the DGE module will find the targeted segment and trim off other parts. Moreover, reads with ambiguous bases will be filtered. (3) The sRNA module incorpates filtering of poly-A tags as polyadenylation is a feature of mRNA data and sRNA sequences can be contaminated by mRNA during sample preparation [42]. (4) The Metagenomics preprocessing module customizes a few functions from the General module for trimming adapters and low-quality bases on both ends, dropping reads with too-short length or too many ambiguous bases. Detailed parameter settings can be accessed in Additional file 3.

### Software features

SOAPnuke is written by C++ for good scalability and performance, and it can be run on both Linux and Windows platforms.

Two paralleled strategies are implemented for acceleration. Multithreading is developed for standalone operation. Data are cut into blocks of fixed size, and each block is processed by 1 thread. This design utilizes multiple cores in a working node. In SOAPnuke, the creation and allocation of threads are managed by a threadpool library, which decreases the overhead of creating and destroying threads. More importantly, Hadoop MapReduce is applied to achieve rapid processing in multinode clusters for ultra-large-scale data. In the mapping phase, each read is kept as a key-value pair, where key is the readID and value denotes the sequence and quality scores. In shuffle phase, the key-value pairs are sorted, and each pair of paired-end reads is gathered. During the reducing phase, blocks of fixed size are processed by various threads of multiple nodes, and each block generates an individual result. After that, it is optional to merge the results into integrated fastq files.

To prove the effectiveness of the acceleration design, we have conducted a performance test on SOAPnuke and other alternative tools. A ∼30× human genome dataset published by GIAB [43] was extracted as testing data (see Additional file 4). In terms of the computing environment, up to 16 nodes were used, each of which has 24 cores of Intel(R) Xeon(R) CPU E5–2620 v4 @ 2.10 GHz and RAM of 128 G. SOAPnuke operations for testing were set as described in published manuscripts (see the reference list in Additional file 5). Trimming adapters and filtering on length and quality were selected for their universality. We chose other workflow-like tools capable of performing these functions, which are Trimmomatic (Trimmomatic, RRID:SCR_011848) [27], AfterQC [30], BBDuk [31], and AlignTrimmer [36]. The parameter setting is also available in Additional file 4.

## Results

In the performance test, we chose 3 indexes for evaluation: elapsed time, CPU usage, and maximum RAM usage. As shown in Table 1, AfterQC is the tool occupying the fewest resources. However, its processing time is too long for practical usage, especially considering that we ran the program with pypy, which is announced to be 3 times as fast as standard Python. Among the remaining tools, SOAPnuke struck an appropriate balance between resource occupancy and performance. Furthermore, users can choose to run SOAPnuke on multiple nodes with MapReduce framework if high-throughput performance is demanded. In our testing, 16 nodes can achieve ∼32 times acceleration compared with standalone operation, which is 5.37 times faster than the highest speed of 4 tested tools.

**Table 1:** Evaluation of the data processing performance across SOAPnuke and 4 other tools

Index\ tools	Time, min	Throughput, reads/s	CPU, %	Max RAM, GB
SOAPnuke (1 node, 1 thread)	302.7	33 947.8	250	0.62
SOAPnuke (16 nodes)	9.4	1 093 191.1	640	50.10
Trimmomatic (1 thread)	84.7	121 380.1	75	2.98
Trimmomatic (24 threads)	50.5	203 582.1	239	10.28
BBDuk	57.2	162 230.2	259	11.40
AlienTrimmer	530.2	19 076.1	99	0.54
AfterQC (pypy)	2482.7	4319.1	99	0.21

Time, throughput, CPU, and maximum memory occupation are presented. For CPU usage, 100% means full load of a single CPU core. Maximum RAM usage means the highest occupancy of RAM during the whole processing.

After the preprocessing, downstream analyses were performed with the GATK (GATK, RRID:SCR_001876) best practice pipeline (see the description of GATK best practices) [44]. Data were processed by the alignment, rmDup, baseRecal, bamSort, and haplotypeCaller modules in order. For the haplotypeCaller, GIAB high-confidence small variant and reference calls v3.3.2 [45] were used as gold standard. Details of this testing are available in Additional file 4.

As seen in Table 2, AfterQC achieves the best variant calling result. The F-measures of SOAPnuke and Trimmomatic are the same, which are slightly lower than those of AfterQC. AlienTrimmer performs slightly worse, and BBDuk has the worst result, whose INDEL calling result differs greatly from that of other tools. In summary, though the variant calling result of AfterQC is optimal, it is not worth considering for its long processing time. Among the remaining tools, SOAPnuke and Trimmomatic tie for first place.

**Table 2:** Variant calling result of SOAPnuke and other 4 tools

Indexes Tools	SNPs precision	SNPs sensitivity	SNPs F-measure	INDELs precision	INDELs sensitivity	INDELs F-measure
SOAPnuke	0.9967	0.9811	0.9888	0.9806	0.9575	0.9689
Trimmomatic	0.9966	0.9811	0.9888	0.9806	0.9575	0.9689
BBDuk	0.9966	0.9797	0.9881	0.9698	0.9184	0.9434
AlienTrimmer	0.9954	0.9810	0.9882	0.9792	0.9540	0.9665
AfterQC	0.9968	0.9811	0.9889	0.9811	0.9586	0.9697

F-measure is a measure considering both the precision and recall of the variant calling result. SNP and INDEL are 2 main categories of variants.

## Discussion and Conclusion

Data quality is critical to downstream analysis, which makes it important to use reliable tools for preprocessing. To omit unnecessary input/output and computation, workflow-like structure is adopted in SOAPnuke, where QC and preprocessing functions are integrated within an executable program. Compared with most of workflow-like tools, such as PrinSeq [6] and RObiNA [26], SOAPnuke adds statistics of preprocessed data for better understanding of data. To cope with datasets generated from different experiments, 4 modules are predefined with tailored functions and parameters. In terms of acceleration approach, multithreading is the sole method adopted by existing tools [14–16, 24–28], but it is only applicable to single-node operations. SOAPnuke utilizes MapReduce to realize concurrent execution on multinode operations, where CPU cores of multiple nodes can be involved in a single task. It improves the scalability of parallel execution and the applicability to mass data. SOAPnuke also includes multithreading for standalone computing. Our test results indicate that SOAPnuke can achieve a speed ∼5.37 times faster than the maximum speed of other tools with multithreading. It is worth mentioning that processing speed is not directly proportional to the number of working nodes, because some procedures like initialization of MapReduce cannot be accelerated as nodes increase, and the burden of communication between nodes aggravates as well.

For the future works, we will continue adding functions to feature modules. For example, in the preprocessing of DGE datasets, filtering out singleton reads is frequently included [46–48]. For the sRNA module, screening out reads based on alignment with noncoding RNA databases (such as tRNA, rRNA, and snoRNA) [49, 50] is under development. Adding statistics such as per-read quality distribution and length distribution is also worth consideration. To users without a computing cluster, SOAPnuke might not be an optimal tool in terms of overall performance. Thus, we are performing refactoring to increase the standalone processing speed.

However, we have found 2 problems worth exploring regarding QC and preprocessing. First, in terms of preprocessing, it is difficult to choose optimal parameters for a specific dataset. Datasets from the same experiments and sequencers tend to share features, so users always select the same parameters for those similar data. The parameters are initially defined based on experiments on a specific dataset or just experience, which may already introduce some error and bias. Moreover, even if the parameters are optimal for the tested dataset, they are possibly inappropriate for other data because of random factors. Thus, the current method is a compromise. However, it might be a considerable solution that preprocessing settings are automatically adjusted during the processing. Second, some of the QC statistics are of limited help to judge the availability of data. For example, as the threshold of filtering out low-quality reads is increased from 0 to 40, the mean quality of all reads or each position will rise accordingly, and the result of variant calling will be improved at the very beginning but then gets worse. This is because preprocessing is a procedure required to strike a balance between removing noise and keeping useful information, while single QC statistics cannot reflect the global balance. A comprehensive list of QC statistics in SOAPnuke can help solve the problem as raising the threshold of mean quality after the balance alone might make other irrelevant statistics worse. Thus, it is worthwhile to explore ways to comprehensively analyze all statistics to evaluate the effect of preprocessing. Currently, this procedure is performed empirically by users. In our future work, these 2 problems will be considered for the development of updated versions.

## Availability and requirements

Project name: SOAPnuke

Project home page: https://github.com/BGI-flexlab/SOAPnuke

RRID:SCR_015025

Operating system(s): Linux, Windows

Programming language: C++

Requirements: libraries: boost, zlib, log4cplus, and openssl; R

License: GPL

## Availability of supporting data

Snapshots of the code and test data are also stored in the *GigaScience* repository, *Giga*DB [51].

## Abbreviations

DGE: digital gene expression; HTS: high-throughput sequencing; QC: quality control; sRNA: small RNA.

## Author contributions

L.F. and Q.C. conceived the project. Yuxin C. and C.S. conducted the survey on existing tools for QC and preprocessing. Yuxin C., Yongsheng C., C.S., Z.H., Y.Z., S.L., J.Y., Z.L., X.Z., J.W., H.Y., L.F., and Q.C., provided feedback on features and functionality. YongSheng C., Z.H., and S.L. wrote the standalone version of SOAPnuke. Yuxin C. wrote the MapReduce version of SOAPnuke. Yuxin C. and Z.H. performed the above-mentioned test. Yuxin C., Y.L., C.Y., and L.F. wrote the manuscript. All authors read and approved the final manuscript.

## Additional files

Supplementary Material 1: Comparison of features and functions of various tools for QC and preprocessing (XLSX 41 kb).

Supplementary Material 2: Details of QC in SOAPnuke (PDF 304 kb).

Supplementary Material 3: Details of preprocessing in SOAPnuke (PDF 1.6 mb).

Supplementary Material 4: Details of preprocessing performance test and downstream analyses (DOCX 38 kb).

Supplementary Material 5: Details of research involving SOAPnuke (XLSX 12 kb).

## Competing interests

The authors declare that they have no competing interests.

## Open access

This article is distributed under the terms of the Creative Commons Attribution 4.0 International License (http://creativecommons.org/licenses/by/4.0/), which permits unrestricted use, distribution, and reproduction in any medium, provided you give appropriate credit to the original author(s) and the source, provide a link to the Creative Commons license, and indicate if changes were made. The Creative Commons Public Domain Dedication waiver (http://creativecommons.org/publicdomain/zero/1.0/) applies to the data made available in this article, unless otherwise stated.

## Supplementary Material

GIGA-D-17-00173_Original_Submission.pdf

GIGA-D-17-00173_Revision_1.pdf

Response_to_Reviewer_Comments_Original_Submission.pdf

Reviewer_1_Report_(Original_Submission) -- Joshua W. K. Ho 03 Aug 2017 Reviewed

Supplement materials

## References

[1] Fox S, Filichkin S, Mockler TC Applications of ultra-high-throughput sequencing. Methods Mol Biol 2009;553:79–108. 19588102 10.1007/978-1-60327-563-7_5

[2] Soon WW, Hariharan M, Snyder MP High-throughput sequencing for biology and medicine. Mol Syst Biol 2014;9(1):640-. 10.1038/msb.2012.61 PMC3564260 23340846

[3] Stephens ZD, Lee SY, Faghri F Big data: astronomical or genomical? PLoS Biol 2015;13(7):e1002195. 26151137 10.1371/journal.pbio.1002195 PMC4494865

[4] Guo Y, Ye F, Sheng Q Three-stage quality control strategies for DNA re-sequencing data. Brief Bioinformatics 2014;15(6):879–89. 24067931 10.1093/bib/bbt069 PMC4492405

[5] Zhou X, Rokas A Prevention, diagnosis and treatment of high-throughput sequencing data pathologies. Mol Ecol 2014;23(7):1679–700. 24471475 10.1111/mec.12680

[6] Schmieder R, Edwards R Quality control and preprocessing of metagenomic datasets. Bioinformatics 2011;27(6):863–4. 21278185 10.1093/bioinformatics/btr026 PMC3051327

[7] Moxon S, Schwach F, Dalmay T A toolkit for analysing large-scale plant small RNA datasets. Bioinformatics 2008;24(19):2252–3. 18713789 10.1093/bioinformatics/btn428

[8] Gordon A, Hannon GJ Fastx-toolkit. FASTQ/A short-reads preprocessing tools. http://hannonlab.cshl.edu/fastx_toolkit. Accessed 1 November 2017.

[9] Cox MP, Peterson DA, Biggs PJ SolexaQA: At-a-glance quality assessment of Illumina second-generation sequencing data. BMC Bioinformatics 2010;11(1):485. 20875133 10.1186/1471-2105-11-485 PMC2956736

[10] Zhang T, Luo Y, Liu K BIGpre: a quality assessment package for next-generation sequencing data. Genomics Proteomics Bioinformatics 2011;9(6):238–44. 22289480 10.1016/S1672-0229(11)60027-2 PMC5054156

[11] Aronesty E ea-utils: Command-Line Tools for Processing Biological Sequencing Data. Durham, NC: Expression Analysis; 2011.

[12] Yang X, Liu D, Liu F HTQC: a fast quality control toolkit for Illumina sequencing data. BMC Bioinformatics 2013;14(1):33. 23363224 10.1186/1471-2105-14-33 PMC3571943

[13] Li H seqtk: toolkit for processing sequences in FASTA/Q formats. https://github.com/lh3/seqtk. Accessed 1 March 2017.

[14] Zhou Q, Su X, Wang A QC-Chain: fast and holistic quality control method for next-generation sequencing data. PLoS One 2013;8(4):e60234. 23565205 10.1371/journal.pone.0060234 PMC3615005

[15] Zhou Q, Su X, Jing G Meta-QC-Chain: comprehensive and fast quality control method for metagenomic data. Genomics Proteomics Bioinformatics 2014;12(1):52–56. 24508279 10.1016/j.gpb.2014.01.002 PMC4411374

[16] Patel RK, Jain M NGS QC Toolkit: a toolkit for quality control of next generation sequencing data. PLoS One 2012;7(2):e30619. 22312429 10.1371/journal.pone.0030619 PMC3270013

[17] Simon A FastQC: a quality control tool for high throughput sequence data. http://www.bioinformatics.babraham.ac.uk/projects/fastqc/ Accessed 1 November 2017.

[18] Schmieder R, Lim YW, Rohwer F TagCleaner: identification and removal of tag sequences from genomic and metagenomic datasets. BMC Bioinformatics 2010;11(1):341. 20573248 10.1186/1471-2105-11-341 PMC2910026

[19] Falgueras J, Lara AJ, Fernandez-Pozo N SeqTrim: a high-throughput pipeline for preprocessing any type of sequence reads. BMC Bioinformatics 2010;11(1):38. 20089148 10.1186/1471-2105-11-38 PMC2832897

[20] St John J SeqPrep: tool for stripping adaptors and/or merging paired reads with overlap into single reads. https://github.com/jstjohn/SeqPrep Accessed 1 November 2017.

[21] Kong Y Btrim: a fast, lightweight adapter and quality trimming program for next-generation sequencing technologies. Genomics 2011;98(2):152–3. 21651976 10.1016/j.ygeno.2011.05.009

[22] Lohse M, Bolger AM, Nagel A RobiNA: a user-friendly, integrated software solution for RNA-seq-based transcriptomics. Nucleic Acids Res 2012;40(W1):W622–7. 22684630 10.1093/nar/gks540 PMC3394330

[23] Martin M Cutadapt removes adapter sequences from high-throughput sequencing reads. EMBnet J 2011;17(1):pp–10.

[24] Schubert M, Lindgreen S, Orlando L AdapterRemoval v2: rapid adapter trimming, identification, and read merging. BMC Res Notes 2016;9(1):88. 26868221 10.1186/s13104-016-1900-2 PMC4751634

[25] Dodt M, Roehr JT, Ahmed R FLEXBAR-flexible barcode and adapter processing for next-generation sequencing platforms. Biology (Basel) 2012;1(3):895–905. 24832523 10.3390/biology1030895 PMC4009805

[26] Li YL, Weng JC, Hsiao CC PEAT: an intelligent and efficient paired-end sequencing adapter trimming algorithm. BMC Bioinformatics 2015;16(Suppl 1):S2. 10.1186/1471-2105-16-S1-S2 PMC4331701 25707528

[27] Bolger AM, Lohse M, Usadel B Trimmomatic: a flexible trimmer for Illumina sequence data. Bioinformatics 2014;30(15):2114–20. 24695404 10.1093/bioinformatics/btu170 PMC4103590

[28] Sturm M, Schroeder C, Bauer P SeqPurge: highly-sensitive adapter trimming for paired-end NGS data. BMC Bioinformatics 2016;17(1):208. 27161244 10.1186/s12859-016-1069-7 PMC4862148

[29] Jiang H, Lei R, Ding SW Skewer: a fast and accurate adapter trimmer for next-generation sequencing paired-end reads. BMC Bioinformatics 2014;15(1):182. 24925680 10.1186/1471-2105-15-182 PMC4074385

[30] Chen S, Huang T, Zhou Y AfterQC: automatic filtering, trimming, error removing and quality control for fastq data. BMC Bioinformatics 2017;18(S3):80. 28361673 10.1186/s12859-017-1469-3 PMC5374548

[31] BUSHNELL Brian BBMap: A Fast, Accurate, Splice-Aware Aligner. Berkeley, CA: Ernest Orlando Lawrence Berkeley National Laboratory; 2014.

[32] Joshi NA, Fass JN Sickle: A sliding-window, adaptive, quality-based trimming tool for FastQ files. https://github.com/najoshi/sickle. Accessed 1 November 2017.

[33] Pertea G fqtrim: trimming&filtering of next-gen reads. https://ccb.jhu.edu/software/fqtrim/. Access 1 November 2017.

[34] Vince B Scythe: a Bayesian adapter trimmer. https://github.com/vsbuffalo/scythe Access 1 March 2017.

[35] Leggett RM, Clavijo BJ, Clissold L NextClip: an analysis and read preparation tool for Nextera long mate pair libraries. Bioinformatics 2014;30(4):566–8. 24297520 10.1093/bioinformatics/btt702 PMC3928519

[36] Criscuolo A, Brisse S AlienTrimmer: a tool to quickly and accurately trim off multiple short contaminant sequences from high-throughput sequencing reads. Genomics 2013;102(5–6):500–6. 23912058 10.1016/j.ygeno.2013.07.011

[37] Goecks J, Nekrutenko A, Taylor J Galaxy: a comprehensive approach for supporting accessible, reproducible, and transparent computational research in the life sciences. Genome Biol 2010;11(8):R86. 20738864 10.1186/gb-2010-11-8-r86 PMC2945788

[38] Team RC R: A Language and Environment for Statistical Computing. Vienna, Austria: R Foundation for Statistical Computing; 2013.

[39] Illumina NextSeq 500 system overview. https://support.illumina.com/content/dam/illumina-support/courses/nextseq-system-overview/story_content/external_files/NextSeq500_System_Overview_narration.pdf Accessed 1 November 2017.

[40] Huang J, Liang X, Xuan Y A reference human genome dataset of the BGISEQ-500 sequencer. Gigascience 2017;6(5):1–9. 10.1093/gigascience/gix024 PMC5467036 28379488

[41] Zhang X, Hao L, Meng L Digital gene expression tag profiling analysis of the gene expression patterns regulating the early stage of mouse spermatogenesis. PLoS One 2013;8(3):e58680. 23554914 10.1371/journal.pone.0058680 PMC3598852

[42] Tam S, Tsao MS, McPherson JD Optimization of miRNA-seq data preprocessing. Brief Bioinformatics 2015;16(6):950–63. 25888698 10.1093/bib/bbv019 PMC4652620

[43] Zook JM, Catoe D, McDaniel J Extensive sequencing of seven human genomes to characterize benchmark reference materials. Sci Data 2016;3:160025. 27271295 10.1038/sdata.2016.25 PMC4896128

[44] GATK best practices http://www.broadinstitute.org/gatk/guide/best-practices. Access 1 November 2017.

[45] NISTv3.3.2, NA12878 high-confidence variant calls as a gold standard GIAB. 2017 ftp://ftp-trace.ncbi.nlm.nih.gov/giab/ftp/release/NA12878_HG001/NISTv3.3.2/. Access 1 November 2017.

[46] Zhang X, Hao L, Meng L Digital gene expression tag profiling analysis of the gene expression patterns regulating the early stage of mouse spermatogenesis. PLoS One 2013;8(3):e58680. 23554914 10.1371/journal.pone.0058680 PMC3598852

[47] Zhou L, Chen J, Li Z Integrated profiling of microRNAs and mRNAs: microRNAs located on Xq27.3 associate with clear cell renal cell carcinoma. PLoS One 2010;5(12):e15224. 21253009 10.1371/journal.pone.0015224 PMC3013074

[48] Han Y, Zhang X, Wang W The suppression of WRKY44 by GIGANTEA-miR172 pathway is involved in drought response of Arabidopsis thaliana. PLoS One 2013;8(11):e73541. 24223111 10.1371/journal.pone.0073541 PMC3819348

[49] Hall AE, Lu WT, Godfrey JD The cytoskeleton adaptor protein ankyrin-1 is upregulated by p53 following DNA damage and alters cell migration. Cell Death Dis 2016;7(4):e2184. 27054339 10.1038/cddis.2016.91 PMC4855670

[50] Surbanovski N, Brilli M, Moser M A highly specific microRNA-mediated mechanism silences LTR retrotransposons of strawberry. Plant J 2016;85(1):70–82. 26611654 10.1111/tpj.13090

[51] Chen Y, Chen Y, Shi C Supporting data for “SOAPnuke: a MapReduce acceleration-supported software for integrated quality control and preprocessing of high-throughput sequencing data.” GigaScience Database 2017 http://dx.doi.org/10.5524/100373.

