# Supplementary material for: SOAPnuke: a MapReduce acceleration-supported software for integrated quality control and preprocessing of high-throughput sequencing data
Source: Gigascience. 2017 Dec 4;7(1):gix120. doi: 10.1093/gigascience/gix120 (PMC5788068; doi:10.1093/gigascience/gix120)
Supplement: GIGA-D-17-00173_Revision_1.pdf [file gix120_giga-d-17-00173_revision_1.pdf]

GigaScience

SOAPnuke: A MapReduce Acceleration supported Software for integrated Quality Control and Preprocessing of High-Throughput Sequencing Data

--Manuscript Draft--

Manuscript Number:	GIGA-D-17-00173R1
Full Title:	SOAPnuke: A MapReduce Acceleration supported Software for integrated Quality Control and Preprocessing of High-Throughput Sequencing Data
Article Type:	Technical Note
Funding Information:	Science & Technology Bureau of Fujian Province (2013YZ0002-2) — Mr. Lin Fang<br>the Joint Project of Natural Science and health Foundation of Fujian Province (2015J01397) — Ms. Chunmei Shi
Abstract:	Quality Control (QC) and preprocessing are essential steps for sequencing data analysis to ensure the accuracy of results. However, existing tools cannot provide a satisfying solution with integrated comprehensive functions, proper architectures and highly-scalable acceleration. In this article, we demonstrate SOAPnuke as a tool with abundant functions for a 'QC-Preprocess-QC' workflow and MapReduce acceleration framework. Four modules with different preprocessing functions are designed for processing datasets from genomic, small RNA (sRNA), Digital Gene Expression (DGE) and metagenomic experiments respectively. As a workflow-like tool, SOAPnuke centralizes processing functions in one executable and predefine their order to avoid the necessity of reformatting different files when switching tools. Furthermore, the MapReduce framework enables large scalability to distribute all the processing works to an entire compute cluster.<br>We conducted a benchmarking where SOAPnuke and other tools are used to preprocess ~30x NA12878 dataset published by GIAB. The standalone operation of SOAPnuke struck a balance between resource occupancy and performance. When accelerated on 16 working nodes with MapReduce, SOAPnuke achieved ~5.7 times of the fastest speed of other tools.
Corresponding Author:	Lin Fang<br><br>CHINA
Corresponding Author Secondary Information:	
Corresponding Author's Institution:	
Corresponding Author's Secondary Institution:	
First Author:	Yuxin Chen
First Author Secondary Information:	
Order of Authors:	Yuxin Chen
	Yongsheng Chen
	Chunmei Shi
	Zhibo Huang
	Yong Zhang
	Shengkang Li
	Yan Li
	Jia Ye
	Chang Yu

Powered by Editorial Manager® and ProduXion Manager® from Aries Systems Corporation

	Zhuo Li
	Xiuqing Zhang
	Jian Wang
	Huanming Yang
	Lin Fang
	Qiang Chen
**Order of Authors Secondary Information:**	
**Response to Reviewers:**	Dear Reviewer:<br><br>Thank you for your letter. We were pleased to know that our manuscript was rated as potentially acceptable for publication in GigaScience, subject to adequate revision and response to your comments.<br><br>We revised the manuscript following the instructions provided in your letter. To address the first question, two sentences has been added to the DISCUSSION section: 'To users without computing cluster, SOAPnuke might not be an optimal tool in terms of overall performance. Thus, we are performing refactoring to increase the standalone processing speed'. As suggested in the second question, Variant calling results and corresponding description have been added to RESULT section. We also greatly appreciate the suggestions offered in third question and have changed the wording and bottom-half content. In terms of language editing, we have fixed the problems you indicated and re-collated the whole manuscript.<br><br>We would like to express our sincere gratitude to you for improving the quality of our manuscript with helpful suggestions.<br><br>Thank you.<br><br>Best Regards,<br>Magic Fang
**Additional Information:**	
**Question**	**Response**
Are you submitting this manuscript to a special series or article collection?	No
**Experimental design and statistics**<br><br>Full details of the experimental design and statistical methods used should be given in the Methods section, as detailed in our Minimum Standards Reporting Checklist. Information essential to interpreting the data presented should be made available in the figure legends.<br><br>Have you included all the information requested in your manuscript?	Yes
**Resources**<br><br>A description of all resources used, including antibodies, cell lines, animals and software tools, with enough information to allow them to be uniquely	Yes

identified, should be included in the Methods section. Authors are strongly encouraged to cite Research Resource Identifiers (RRIDs) for antibodies, model organisms and tools, where possible.<br><br>Have you included the information requested as detailed in our Minimum Standards Reporting Checklist?	
**Availability of data and materials**<br><br>All datasets and code on which the conclusions of the paper rely must be either included in your submission or deposited in publicly available repositories (where available and ethically appropriate), referencing such data using a unique identifier in the references and in the "Availability of Data and Materials" section of your manuscript.<br><br>Have you have met the above requirement as detailed in our Minimum Standards Reporting Checklist?	Yes

Manuscript
Click here to download Manuscript manuscript(SOAPnuke SCE)(1).docx

Technical Note:

SOAPnuke: A MapReduce Acceleration supported Software for integrated Quality Control and Preprocessing of High-Throughput Sequencing Data

Yuxin Chen[1†], Yongsheng Chen[2†], Chunmei Shi[3,4,5†], Zhibo Huang[1], Yong Zhang[1,6], Shengkang Li[1,6], Yan Li[1], Jia Ye[1], Chang Yu[7], Zhuo Li[8,9], Xiuqing Zhang[1], Jian Wang[1,10], Huanming Yang[1,10], Lin Fang[1,6] and Qiang Chen[3,4,5]***

1 BGI-Shenzhen, Shenzhen 518083; 2 Geneplus-Beijing, Beijing 102206; 3 Department of Oncology, Fujian Medical University Union Hospital, Fuzhou 350001; 4 Fujian Key Laboratory of Translational Cancer Medicine, Fuzhou 350014; 5 Department of Stem Cell Research Institute, Fujian Medical University Stem Cell Research Institute, Fuzhou 350000; 6 Collaborative Innovation Center of High Performance Computing, National University of Defense Technology, Changsha 410073; 7 Intel China Ltd., Shanghai 200336; 8 Guangdong Provincial Hospital of Chinese Medicine, Guangzhou 510120; 9 Department of Surgery, Faculty of Medicine, The Chinese University of Hong Kong, Hong Kong; 10 James D. Watson Institute of Genome Sciences, Hangzhou 310058, China

Yuxin C chenyuxin@genomics.cn, ORCID: 0000-0002-9246-1829; Yongsheng C chenys@geneplus.org.cn; CS scmfz@qq.com; ZH huangzhibo@genomics.cn, ORCID: 0000-0002-2750-6517; YZ zhangyong2@genomics.cn; SL lishengkang@genomics.cn, ORCID: 0000-0002-6864-5644; YL liyan@genomics.cn; JY yejia@genomics.cn; CY chang.yu@intel.com;ZL lzgdphcm@163.com; XZ zhangxq@genomics.cn; JW wangjian@genomics.cn;HY yanghm@genomics.cn, ORCID: 0000-0002-0858-3410; LF fangl@genomics.cn, ORCID: 0000-0002-5954-3435; QC cqiang8@189.cn

[†]Contributed equally

*First corresponding author

**Second corresponding author

ABSTRACT

Quality Control (QC) and preprocessing are essential steps for sequencing data analysis to ensure the accuracy of results. However, existing tools cannot provide a satisfying solution with integrated comprehensive functions, proper architectures and highly-scalable acceleration. In this article, we demonstrate SOAPnuke as a tool with abundant functions for a 'QC-Preprocess-QC' workflow and MapReduce acceleration framework. Four modules with different preprocessing functions are designed for processing datasets from genomic, small RNA

(sRNA), Digital Gene Expression (DGE) and metagenomic experiments respectively. As a workflow-like tool, SOAPnuke centralizes processing functions in one executable and predefine their order to avoid the necessity of reformatting different files when switching tools. Furthermore, the MapReduce framework enables large scalability to distribute all the processing works to an entire compute cluster.

We conducted a benchmarking where SOAPnuke and other tools are used to preprocess ~30x NA12878 dataset published by GIAB. The standalone operation of SOAPnuke struck a balance between resource occupancy and performance. When accelerated on 16 working nodes with MapReduce, SOAPnuke achieved ~5.7 times of the fastest speed of other tools.

KEYWORDS: High-throughput sequencing, Quality control, Preprocessing, MapReduce

BACKGROUND

High-throughput sequencing (HTS) instruments have enabled many large-scale studies and generated enormous amount of data [1-3]. However, the presence of low-quality bases, sequence artifacts and sequence contamination can introduce serious negative impact on downstream analyses. Thus, QC and preprocessing of raw data serve as the critical steps to initiate analysis pipelines [4, 5]. QC investigates several statistics of datasets to ensure data quality, and preprocessing trims off undesirable terminal fragments and filters out substandard reads [6]. We have conducted a survey on existing 31 tools and widely shared functions are listed in Supplementary Material 1.

Existing tools for QC and preprocessing can be divided into two categories according to their structures: toolkit and workflow. Toolkit-like software provides multiple executables such as statistics computer, clipper and filtrator [7-15]. In practice, raw data is processed by a few

individual executables in sequence. Comparatively, workflow-like software offers an integral workflow where functions are performed in predefined order [6, 16-27].

However, both categories have their own demerits. When using toolkit-like software, it is complex and error-prone to write additional scripts to wrap executables. Moreover, it consumes much time to generate and read intermediate files, which is hard for acceleration. Besides, the same variables could possibly be computed repetitively. For instance, average quality score of each read is necessary for counting quality score distribution by reads, and filtering reads based on average quality scores. It has to be counted twice if these two functions are implemented by different toolkits.

For workflow-like tools, an optimal architecture is required since the orders of functions are fixed. Most of existing tools successively perform QC and preprocessing without complete statistics of preprocessed datasets. If the preprocessing operation is not suitable for a given dataset, the problem can only be revealed by downstream analyses.

Datasets sequenced from various samples may require different processing functions or parameters. Existing workflow-like tools mostly support genomics data processing, only a few of them are developed for other types of studies, such as RNA-seq and metagenomics data. For example, RObiNA [22] provides four modules for different RNA sequencing experiments. PrinSeq [6] offers a QC stat, dinucleotide odds ratios, to show how the dataset might be related to other viral/microbial metagenomes. However, there is still no single tool supporting multiple data types.

Several tools have made certain progress in overcoming the limitations mentioned above. Galaxy [37] is a web-based platform incorporating various existing toolkit-like software. Users can conveniently concatenate tools into a pipeline on the web interface. NGS QC toolkit [16]

offers a workflow with QC on both raw and preprocessed datasets, though the preprocessing functions are too few.

In terms of software acceleration, only multi-threading is adopted by existing tools [14-16, 24-28]. This approach only works for standalone operation and is limited by the maximum number of processors in one computer server. It may be incompetent when dealing with huge present and potential volume of sequencing datasets.

To solve these problems, we have developed a workflow-like tool, SOAPnuke, for integrated QC and preprocessing of large HTS datasets. Similar to NGS QC toolkit, SOAPnuke performs two-step QC. Trimming, filtering and other frequently used functions are integrated in our program. Four modules are designed to handle genomic, metagenomic, DGE and sRNA datasets respectively. In addition, SOAPnuke is extended to multiple working nodes for parallel computing using Hadoop MapReduce framework.

METHODS

QC & PREPROCESSING

SOAPnuke (SOAPnuke, RRID:SCR_015025) was developed to summarize statistics of both raw and preprocessed data. Basic statistics are comprised of the number of sequences and bases, base composition, Q20 and Q30, and filtering information. Complex statistics include the distribution of quality score and base composition distribution for each position. For the quality score distribution, Q20 and Q30 for each position are plotted in line chart and the quantiles of the quality are represented in a boxplot. And for the base composition distribution, an overlapping histogram is used to display base composition distribution for each position. These calculations are conducted by C++ and the plots are generated by R 3.3.2 [38]. An example of the two plots are shown in Fig.1. A comprehensive list of statistics available in SOAPnuke is

included in Additional File 2. Statistics of preprocessed data are compared with some preset thresholds. A warning message will be issued if median score of any position in per-base quality distribution is lower than 25 and a failure will be issued if lower than 20. For per-base base composition, a warning will be raised if difference between A and T, or G and C in any position is greater than 10% or a failure will be issued if greater than 20%.

Fig.1. An example of QC complex statistics. (a) per-base quality distribution of raw paired-end reads. (b) per-base Q20 and Q30 of raw and preprocessed paired-end reads. (c) per-base base composition distribution of raw paired-end reads.

In the step of preprocessing, those undesirable terminal fragments are trimmed off, substandard reads are filtered out, and certain transform operations are applied. On both ends of reads, bases of assigned number or of quality lower than threshold will be trimmed off. Sequencing adapters can be aligned, where mismatch is supported while no INDEL is tolerated, and cut to 3' end. Filtering can be performed on reads with adapter, short length, too many ambiguous bases, low average quality or too many low-quality bases. The sequencing batches, such as tile of Illumina sequencer[39] and fov (field of view) of BGI sequencer[40], with unfavorable sequencing quality can be assigned so the corresponding sequences will be discarded. In addition, reads with identical nucleotides can be deduplicated to keep only one copy. Transformation comprises quality system conversion, interconversion between DNA and RNA, and compression of output with gzip, etc. Additional File 3 lists the above preprocessing functions and their parameters.

MODULES DESIGN

To improve processing performance of different types of data, four modules are specialized in SOAPnuke, including General, DGE, sRNA and Meta modules. (1) General module can handle most of the DNA re-sequencing datasets, as described in the section of QC & PROCESSING. (2)

DGE Profiling generates single-end read which has a 'CATG' segment neighboring targeted sequences of 17 base pairs[41]. By default, DGE module will find the targeted segment and trim off other parts. Moreover, reads with ambiguous bases will be filtered. (3) sRNA module incorpates filtering of poly-A tags as polyadenylation is a feature of mRNA data and sRNA sequences can be contaminated by mRNA during sample preparation[42]. (4) Metagenomics preprocessing module customizes a few functions from General module for trimming adapters and low-quality bases on both ends, dropping reads with too short length or too many ambiguous bases. Detailed parameters settings can be accessed in Additional File 3.

SOFTWARE FEATURES

SOAPnuke is written by C++ for good scalability and performance and it can be run on both Linux and Windows platforms.

Two paralleled strategies are implemented for acceleration. Multi-threading is developed for standalone operation. Data is cut into blocks of fixed size, and each block is processed by one thread. This design utilizes multiple cores in a working node. In SOAPnuke, the creation and allocation of threads are managed by threadpool library, which decreases the overhead of creating and destroying threads. More importantly, Hadoop MapReduce is applied to achieve rapid processing in multi-node cluster for ultra-large-scale data. In the mapping phase, each read is kept as a key-value pair, where key is readID and value is sequence and quality scores. In shuffle phase, the key-value pairs are sorted, and each pair of paired-end reads is gathered. During the reducing phase, blocks of fixed size are processed by various threads of multiple nodes, and each block generates an individual result. After that, it is optional to merge the results into integrated fastq file(s).

To prove the effectiveness of the acceleration design, we have conducted a performance tests on SOAPnuke and other alternative tools. A ~30x human genome dataset published by GIAB [43]

were extracted as testing data(see Addition File 4) . In terms of computing environment, up to 16 nodes were used, each of which has 24 cores of Intel(R) Xeon(R) CPU E5-2620 v4 @ 2.10GHz and RAM of 128G. SOAPnuke operations for testing were set as described in published manuscripts(see the reference list in Additional File 5). Trimming adapters and filtering on length and quality were selected for their universality. We chose other workflow-like tools capable of performing these functions, which are Trimmomatic (Trimmomatic, RRID:SCR_011848)[27], AfterQC [30], BBDuk [31] and AlignTrimmer [36]. The parameter setting is also available in Addition File 4.

RESULTS

In the performance test, we chose three indexes for evaluation: elapsed time, CPU usage and maximum RAM usage. As shown in Table.1, AfterQC is the tool occupying least resources. However, its processing time is too long for practical usage, especially considering we ran the program with pypy, which is announced to be 3 times as fast as standard python. Among the remaining tools, SOAPnuke struck an appropriate balance between resources occupancy and performance. Furthermore, users can choose to run SOAPnuke on multiple nodes with MapReduce framework if high throughput performance is demanded. In our testing, 16 nodes can achieve ~32 times acceleration compared to standalone operation, which is 5.37 times faster than the highest speed of four tested tools.

Tools \ Indexes	Time (min)	Throughput (read/s)	CPU	Max RAM (GB)
SOAPnuke (1 node 1 thread)	302.7	33947.8	250%	0.62
SOAPnuke (16 nodes)	9.4	1093191.1	640%	50.10
Trimmomatic (1 thread)	84.7	121380.1	75%	2.98
Trimmomatic (24 threads)	50.5	203582.1	239%	10.28
BBDuk	57.2	162230.2	259%	11.40

AlienTrimmer	530.2	19076.1	99%	0.54
AfterQC (pypy)	2482.7	4319.1	99%	0.21

Table.1. Evaluation of the data processing performance across SOAPnuke and four other tools. Time, throughput, CPU and maximum memory occupation are presented. For CPU usage, 100% means full load of a single CPU core. Maximum RAM usage means the highest occupancy of RAM during the whole processing.

After the preprocessing, downstream analyses were performed with GATK (GATK, RRID:SCR_001876) best practice pipeline (see the description of GATK best practices [44]). Data was processed by alignment, rmDup, baseRecal, bamSort and haplotypeCaller modules in order. For the haplotypeCaller, GIAB high-confidence small variant and reference calls v3.3.2 [45] were used as gold standard. Details of this testing is available in Additional File 4.

Indexes / Tools	SNPs Precision	SNPs Sensitivity	SNPs F-measure	INDELs Precision	INDELs Sensitivity	INDELs F-measure
SOAPnuke	0.9967	0.9811	0.9888	0.9806	0.9575	0.9689
Trimmomatic	0.9966	0.9811	0.9888	0.9806	0.9575	0.9689
BBDuk	0.9966	0.9797	0.9881	0.9698	0.9184	0.9434
AlienTrimmer	0.9954	0.9810	0.9882	0.9792	0.9540	0.9665
AfterQC	0.9968	0.9811	0.9889	0.9811	0.9586	0.9697

Table.2. Variant calling result of SOAPnuke and other 4 tools. F-measure is a measure considering both the precision and recall of the variant calling result. SNP and INDEL are two main categories of variants.

As seen in the Table.2, AfterQC achieves best variant calling result. The F-measures of SOAPnuke and Trimmomatic are the same, which are slightly lower than that of AfterQC. AlienTrimmer performs slightly worse, and BBDuk has the worst result whose INDEL calling result differs greatly from that of other tools. In summary, though the variant calling result of AfterQC is optimal, it is not worth considering for its long processing time. Among the remaining tools, SOAPnuke and Trimmomatic tie for first place.

DISCUSSION AND CONCLUSION

Data quality is critical to downstream analysis, which makes it important to use reliable tools for preprocessing. To omit unnecessary input/output and computation, workflow-like structure is adopted in SOAPnuke, where QC and preprocessing functions are integrated within an executable program. Compared to most of workflow-like tools, such as PrinSeq [6] and RObiNA [26], SOAPnuke adds statistics of preprocessed data for better understanding of data. To cope with datasets generated from different experiments, four modules are predefined with tailored functions and parameters. In terms of acceleration approach, multi-threading is the sole method adopted by existing tools [14-16, 24-28] but only applicable to single-node operations. SOAPnuke utilizes MapReduce to realize concurrent execution on multi-node operations, where CPU cores of multiple nodes can be involved in a single task. It improves the scalability of parallel execution and the applicability to mass data. SOAPnuke also include multi-threading for standalone computing. Our test results indicate that SOAPnuke can achieve ~5.37 times faster than the maximum speed of other tools with multi-threading. It is worth mentioning that processing speed is not directly proportional to the number of working nodes, because some procedures like initialization of MapReduce cannot be accelerated as nodes increase, and the burden of communication between nodes aggravates as well.

For the future works, we will continue adding functions to feature modules. For example, in preprocessing of DGE datasets, filtering out singleton reads is frequently included [46-48]. For sRNA module, screening out reads based on alignment with noncoding RNA databases (such as tRNA, rRNA and snoRNA) [49,50] is under development. It is also considerable to add statistics such as per-read quality distribution and length distribution. To users without computing cluster, SOAPnuke might not be an optimal tool in terms of overall performance. Thus, we are performing refactoring to increase the standalone processing speed.

However, we have found two problems worth exploring regarding QC and preprocessing. Firstly, in terms of preprocessing, it is difficult to choose optimal parameters for a specific dataset. Datasets from the same experiments and sequencers tend to share features, so users always select the same parameters for those similar data. The parameters are initially defined based on experiments on a specific dataset or just experience, which may already introduces some error and bias. Moreover, even if the parameters are optimal for the tested dataset, they are possibly inappropriate for other data because of random factors. Thus, the current method is a compromise. However, it might be a considerable solution that preprocessing settings are automatically adjusted during the processing. Secondly, some of the QC statistics are of limited help to judge the availability of data. For example, as the threshold of filtering out low-quality reads is increased from 0 to 40, the mean quality of all reads or each position will rise accordingly, and the result of variant calling will be improved at the very beginning but then gets worse. It is because preprocessing is a procedure required to strike a balance between removing noise and keeping useful information, while single QC statistics cannot reflect the global balance. A comprehensive list of QC statistics in SOAPnuke can help solve the problem since raising the threshold of mean quality after the balance alone might make other irrelevant statistics worse. Thus, it is worthwhile to explore ways to comprehensively analyze all statistics to evaluate the effect of preprocessing. Currently, this procedure is performed empirically by users. In our future work, these two problems will be considered for the development of updated versions.

Availability and requirements

Project name: SOAPnuke

Project home page: https://github.com/BGI-flexlab/SOAPnuke

RRID: SCR_015025

Operating system(s): Linux, Windows

Programming language: C++

Requirements: libraries: boost, zlib, log4cplus and openssl; R

License: GPL

Availability of supporting data

Snapshots of the code and test data are also stored in the *GigaScience* repository, GigaDB [51].

Abbreviations

QC, quality control; HTS, high throughput sequencing; DGE, digital gene expression; sRNA, small RNA

Declarations

ACKNOWLEDGEMENTS

This research was supported by Collaborative Innovation Center of High Performance Computing, Critical Patented Project of the Science & Technology Bureau of Fujian Province, China (Grant No. 2013YZ0002-2) and the Joint Project of Natural Science and health Foundation of Fujian Province, China (Grant No.2015J01397).

AUTHORS' CONTRIBUTIONS

LF and QC conceived the project. Yuxin C and CS conducted the survey on existing tools for QC and preprocessing. Yuxin C, Yongsheng C, CS, ZH, YZ, SL, JY, ZL, XZ, JW, HY, LF, QC provided feedback on features and functionality. YongSheng C, ZH and SL wrote the standalone version of SOAPnuke. Yuxin C wrote the MapReduce version of SOAPnuke. Yuxin C and ZH performed the above-mentioned test. Yuxin C, YL, CY and LF wrote the manuscript. All authors read and approved the final manuscript.

COMPETING INTERESTS

The authors declare that they have no competing interests.

OPEN ACCESS

This article is distributed under the terms of the Creative Commons Attribution 4.0 International License (http://creativecommons.org/licenses/by/4.0/), which permits unrestricted use, distribution, and reproduction in any medium, provided you give appropriate credit to the original author(s) and the source, provide a link to the Creative Commons license, and indicate if changes were made. The Creative Commons Public Domain Dedication waiver (http://creativecommons.org/publicdomain/zero/1.0/) applies to the data made available in this article, unless otherwise stated.

ADDITIONAL FILES

Supplementary Material 1: Comparison of features and functions of various tools for QC and preprocessing. (XLSX 41kb)

Supplementary Material 2: Details of QC in SOAPnuke. (PDF 304kb)

Supplementary Material 3: Details of preprocessing in SOAPnuke. (PDF 1.6mb)

Supplementary Material 4: Details of preprocessing performance test and downstream analyses. (DOCX 38kb)

Supplementary Material 5: Details of researches involving SOAPnuke. (XLSX 12kb)

REFERENCES

1. Fox S, Filichkin S, Mockler TC. **Applications of ultra-high-throughput sequencing**. Methods Mol Biol. 2009;553:79-108.
2. Soon WW, Hariharan M, Snyder MP. **High-throughput sequencing for biology and medicine.** Mol Syst Biol. 2013;9:640.
3. Stephens ZD, Lee SY, Faghri F, Campbell RH, Zhai C, Efron MJ, et al. **Big Data: Astronomical or Genomical?** PLoS Biol. 2015;13(7):e1002195.
4. Guo Y, Ye F, Sheng Q, Clark T, Samuels DC. **Three-stage quality control strategies for DNA re-sequencing data.** Brief Bioinform. 2014;15(6):879-89.
5. Zhou X, Rokas A. **Prevention, diagnosis and treatment of high-throughput sequencing data pathologies.** Mol Ecol. 2014;23(7):1679-700.
6. Schmieder R, Edwards R. **Quality control and preprocessing of metagenomic datasets.** Bioinformatics. 2011;27(6):863-4.
7. Moxon S, Schwach F, Dalmay T, Maclean D, Studholme DJ, Moulton V. **A toolkit for analysing large-scale plant small RNA datasets.** Bioinformatics. 2008;24(19):2252-3.
8. Gordon A, Hannon GJ. **Fastx-toolkit. FASTQ/A short-reads preprocessing tools (unpublished)** http://hannonlab. cshl. edu/fastx_toolkit. Accessed 1 Nov 2017.
9. Cox MP, Peterson DA, Biggs PJ. **SolexaQA: At-a-glance quality assessment of Illumina second-generation sequencing data.** BMC bioinformatics. 2010 Sep 27;11(1):485.

10. Zhang T, Luo Y, Liu K, Pan L, Zhang B, Yu J, et al. **BIGpre: a quality assessment package for next-generation sequencing data.** Genomics Proteomics Bioinformatics. 2011;9(6):238-44.

11. Aronesty E. **ea-utils: Command-line tools for processing biological sequencing data. Expression Analysis**, Durham, NC. 2011.

12. Yang X, Liu D, Liu F, Wu J, Zou J, Xiao X, et al. **HTQC: a fast quality control toolkit for Illumina sequencing data**. BMC Bioinformatics. 2013;14:33.

13. Li H. **seqtk: Toolkit for processing sequences in FASTA/Q formats.** https://github.com/lh3/seqtk. Accessed 1 Mar 2017.

14. Zhou Q, Su X, Wang A, Xu J, Ning K. **QC-Chain: fast and holistic quality control method for next-generation sequencing data**. PLoS One. 2013;8(4):e60234.

15. Zhou Q, Su X, Jing G, Ning K. **Meta-QC-Chain: comprehensive and fast quality control method for metagenomic data.** Genomics Proteomics Bioinformatics. 2014;12(1):52-6.

16. Patel RK, Jain M. **NGS QC Toolkit: a toolkit for quality control of next generation sequencing data.** PLoS One. 2012;7(2):e30619.

17. Simon A. **FastQC: a quality control tool for high throughput sequence data.** http://www.bioinformatics.babraham.ac.uk/projects/fastqc/ Accessed 1 Nov 2017

18. Schmieder R, Lim YW, Rohwer F, Edwards R. **TagCleaner: Identification and removal of tag sequences from genomic and metagenomic datasets.** BMC Bioinformatics. 2010;11:341.

19. Falgueras J, Lara AJ, Fernandez-Pozo N, Canton FR, Perez-Trabado G, Claros MG. **SeqTrim: a high-throughput pipeline for pre-processing any type of sequence read**. BMC Bioinformatics. 2010;11:38.

20. St John, J. **SeqPrep: Tool for stripping adaptors and/or merging paired reads with overlap into single reads.** https://github.com/jstjohn/SeqPrep Accessed 1 Nov 2017

21. Kong Y. **Btrim: a fast, lightweight adapter and quality trimming program for next-generation sequencing technologies.** Genomics. 2011;98(2):152-3.

22. Lohse M, Bolger AM, Nagel A, Fernie AR, Lunn JE, Stitt M, et al. **RobiNA: a user-friendly, integrated software solution for RNA-Seq-based transcriptomics**. Nucleic Acids Res. 2012;40(Web Server issue):W622-7.

23. Martin M. **Cutadapt removes adapter sequences from high-throughput sequencing reads**. EMBnet. journal. 2011 May 2;17(1):pp-10.

24. Schubert M, Lindgreen S, Orlando L. **AdapterRemoval v2: rapid adapter trimming, identification, and read merging.** BMC Res Notes. 2016;9:88.

25. Dodt M, Roehr JT, Ahmed R, Dieterich C. **FLEXBAR-Flexible Barcode and Adapter Processing for Next-Generation Sequencing Platforms**. Biology (Basel). 2012;1(3):895-905.

26. Li YL, Weng JC, Hsiao CC, Chou MT, Tseng CW, Hung JH. **PEAT: an intelligent and efficient paired-end sequencing adapter trimming algorithm**. BMC Bioinformatics. 2015;16 Suppl 1:S2.

27. Bolger AM, Lohse M, Usadel B. **Trimmomatic: a flexible trimmer for Illumina sequence data**. Bioinformatics. 2014;30(15):2114-20.

28. Sturm M, Schroeder C, Bauer P. **SeqPurge: highly-sensitive adapter trimming for paired-end NGS data**. BMC Bioinformatics. 2016;17:208.

29. Jiang H, Lei R, Ding SW, Zhu S. **Skewer: a fast and accurate adapter trimmer for next-generation sequencing paired-end reads**. BMC Bioinformatics. 2014;15:182.

30. Chen S, Huang T, Zhou Y, Han Y, Xu M, Gu J. **AfterQC: automatic filtering, trimming, error removing and quality control for fastq data.** BMC Bioinformatics. 2017;18(Suppl 3):80.

31. BUSHNELL, Brian. **BBMap: A Fast, Accurate, Splice-Aware Aligner.** Ernest Orlando Lawrence Berkeley National Laboratory, Berkeley, CA (US), 2014.

32. Joshi NA, Fass JN. **Sickle: A sliding-window, adaptive, quality-based trimming tool for FastQ files**. https://github.com/najoshi/sickle. Accessed 1 Nov 2017.

33. Pertea, G. **fqtrim: trimming&filtering of next-gen reads.** https://ccb.jhu.edu/software/fqtrim/. Access 1 Nov 2017.

34. Vince B. **Scythe: A Bayesian adapter trimmer**. https://github.com/vsbuffalo/scythe Access 1 Mar 2017.

35. Leggett RM, Clavijo BJ, Clissold L, Clark MD, Caccamo M. **NextClip: an analysis and read preparation tool for Nextera Long Mate Pair libraries.** Bioinformatics. 2014;30(4):566-8.

36. Criscuolo A, Brisse S. **AlienTrimmer: a tool to quickly and accurately trim off multiple short contaminant sequences from high-throughput sequencing reads.** Genomics. 2013;102(5-6):500-6.

37. Goecks J, Nekrutenko A, Taylor J, Galaxy T. **Galaxy: a comprehensive approach for supporting accessible, reproducible, and transparent computational research in the life sciences**. Genome Biol. 2010;11(8):R86.

38. Team RC. **R: A language and environment for statistical computing**. R Foundation for Statistical Computing, Vienna, Austria. 2013.

39. Illumina. NextSeq 500 System Overview. https://support.illumina.com/content/dam/illumina-support/courses/nextseq-system-overview/story_content/external_files/NextSeq500_System_Overview_narration.pdf Accessed 1 Nov 2017.

40. Huang J, Liang X, Xuan Y, Geng C, Li Y, Lu H, Qu S, Mei X, Chen H, Yu T, Sun N. A reference human genome dataset of the BGISEQ-500 sequencer. GigaScience. 2017;6(5):1-9.

41. Zhang X, Hao L, Meng L, Liu M, Zhao L, Hu F, et al. Digital gene expression tag profiling analysis of the gene expression patterns regulating the early stage of mouse spermatogenesis. PLoS One. 2013;8(3):e58680.

42. Tam S, Tsao MS, McPherson JD. Optimization of miRNA-seq data preprocessing. Brief Bioinform. 2015;16(6):950-63.

43. Zook JM, Catoe D, McDaniel J, Vang L, Spies N, Sidow A, et al. **Extensive sequencing of seven human genomes to characterize benchmark reference materials**. Sci Data. 2016;3:160025.

44. GATK best practices http://www.broadinstitute.org/gatk/ guide/best-practices. Access 1st Nov 2017

45. GIAB high-confidence small variant and reference calls v3.3.2. GIAB. 2017 ftp://ftp-trace.ncbi.nlm.nih.gov/giab/ftp/release/NA12878_HG001/NISTv3.3.2/. Access 1 Nov 2017.

46. Zhang X, Hao L, Meng L, Liu M, Zhao L, Hu F, et al. **Digital gene expression tag profiling analysis of the gene expression patterns regulating the early stage of mouse spermatogenesis.** PLoS One. 2013;8(3):e58680.

47. Zhou L, Chen J, Li Z, Li X, Hu X, Huang Y, et al. **Integrated profiling of microRNAs and mRNAs: microRNAs located on Xq27.3 associate with clear cell renal cell carcinoma.** PLoS One. 2010;5(12):e15224.

48. Han Y, Zhang X, Wang W, Wang Y, Ming F. **The suppression of WRKY44 by GIGANTEA-miR172 pathway is involved in drought response of Arabidopsis thaliana**. PLoS One. 2013;8(11):e73541.

49. Hall AE, Lu WT, Godfrey JD, Antonov AV, Paicu C, Moxon S, et al. **The cytoskeleton adaptor protein ankyrin-1 is upregulated by p53 following DNA damage and alters cell migration**. Cell Death Dis. 2016;7:e2184.

50. Surbanovski N, Brilli M, Moser M, Si-Ammour A. **A highly specific microRNA-mediated mechanism silences LTR retrotransposons of strawberry**. Plant J. 2016;85(1):70-82.

51. Chen, Y; Chen, Y; Shi, C; Huang, Z; Zhang, Y; Li, S; Li, Y; Ye, J; Yu, C; Li, Z; Zhang, X; Wang, J; Yang, H; Fang, L; Chen, Q (2017): **Supporting data for "SOAPnuke: A MapReduce Acceleration supported Software for integrated Quality Control and Preprocessing of High-Throughput Sequencing Data**" GigaScience Database. http://dx.doi.org/10.5524/100373

Figure.1

Click here to download Figure Fig.1.pdf

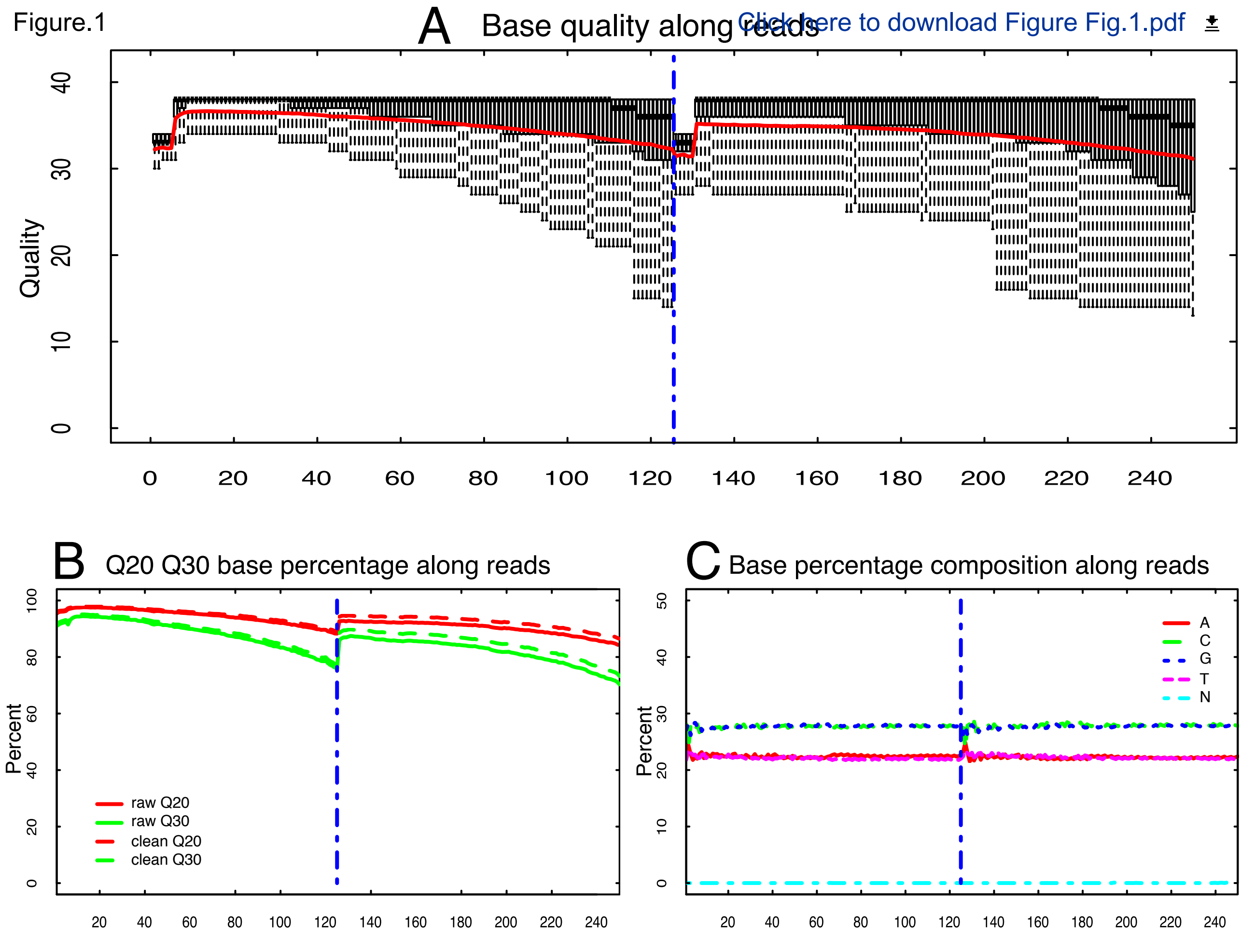

Supplementary Material 1

Click here to access/download
Supplementary Material
SM1.xlsx

Supplementary Material 2

Click here to access/download
Supplementary Material
SM2.pdf

Supplementary Material 3

Click here to access/download
Supplementary Material
SM3.pdf

Click here to access/download
Supplementary Material
SM4.docx

Supplementary Material 5

Click here to access/download
Supplementary Material
SM5.xlsx
