# Supplementary material for: SOAPnuke: a MapReduce acceleration-supported software for integrated quality control and preprocessing of high-throughput sequencing data
Source: Gigascience. 2017 Dec 4;7(1):gix120. doi: 10.1093/gigascience/gix120 (PMC5788068; doi:10.1093/gigascience/gix120)
Supplement: Supplement materials [file gix120_supp.zip › SM3.pdf]

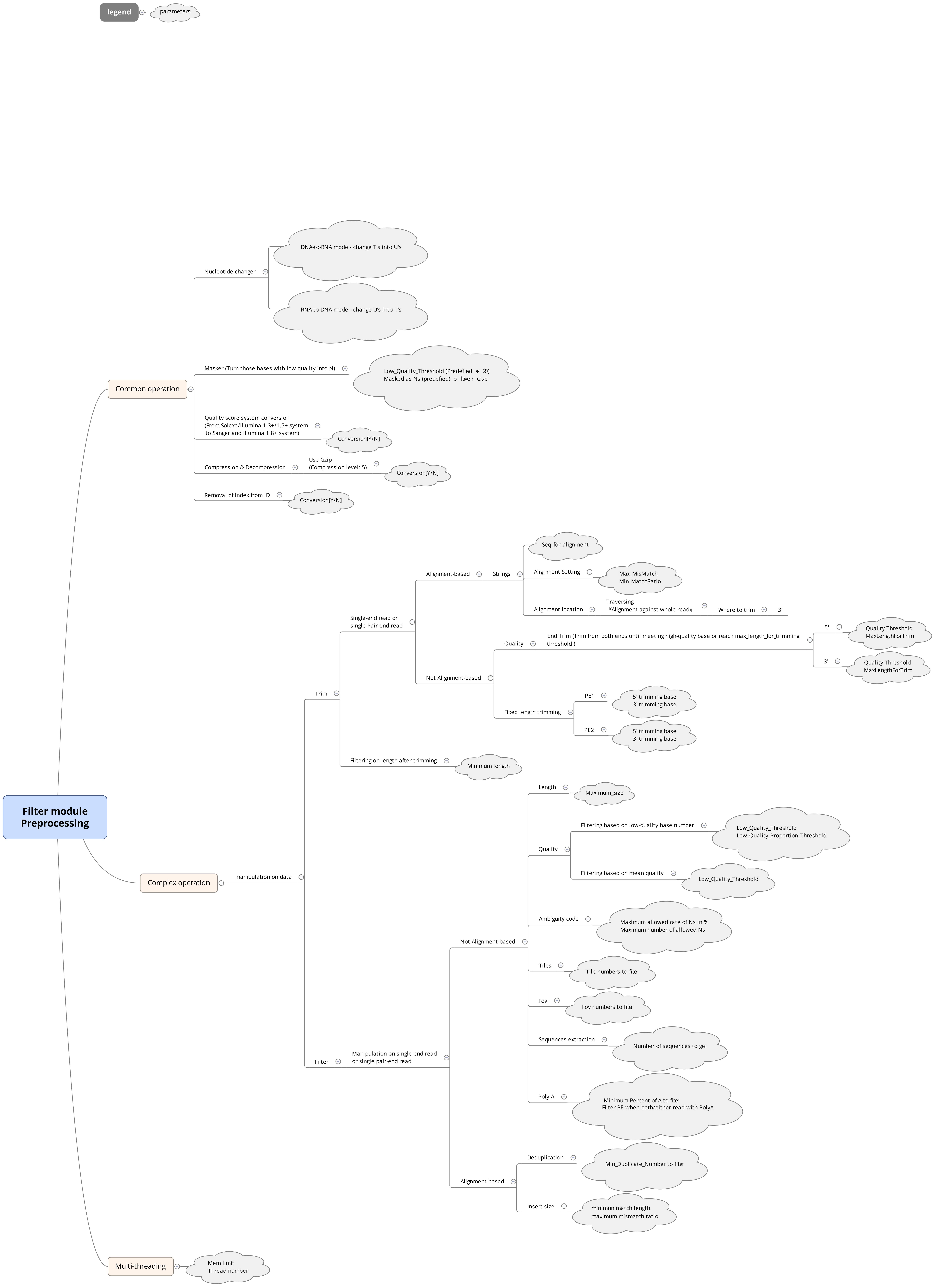

legend
parameters
Filter module Preprocessing
Common operation
Nucleotide changer
DNA-to-RNA mode - change T's into U's
RNA-to-DNA mode - change U's into T's
Masker (Turn those bases with low quality into N)
Low_Quality_Threshold (Predefined as 20)
Masked as Ns (predefined) or lower case
Quality score system conversion
(From Solexa/Illumina 1.3+/1.5+ system
to Sanger and Illumina 1.8+ system)
Conversion[Y/N]
Compression & Decompression
Use Gzip
(Compression level: 5)
Conversion[Y/N]
Removal of index from ID
Conversion[Y/N]
Complex operation
manipulation on data
Trim
Single-end read or
single Pair-end read
Alignment-based
Strings
Seq_for_alignment
Alignment Setting
Max_MisMatch
Min_MatchRatio
Alignment location
Traversing
「Alignment against whole read」
Where to trim
3'
Not Alignment-based
Quality
End Trim (Trim from both ends until meeting high-quality base or reach max_length_for_trimming
threshold)
5'
Quality Threshold
MaxLengthForTrim
3'
Quality Threshold
MaxLengthForTrim
Fixed length trimming
PE1
5' trimming base
3' trimming base
PE2
5' trimming base
3' trimming base
Filtering on length after trimming
Minimum length
Filter
Manipulation on single-end read
or single pair-end read
Not Alignment-based
Length
Maximum_Size
Quality
Filtering based on low-quality base number
Low_Quality_Threshold
Low_Quality_Proportion_Threshold
Filtering based on mean quality
Low_Quality_Threshold
Ambiguity code
Maximum allowed rate of Ns in %
Maximum number of allowed Ns
Tiles
Tile numbers to filter
Fov
Fov numbers to filter
Sequences extraction
Number of sequences to get
Poly A
Minimum Percent of A to filter
Filter PE when both/either read with PolyA
Alignment-based
Deduplication
Min_Duplicate_Number to filter
Insert size
minimun match length
maximum mismatch ratio
Multi-threading
Mem limit
Thread number

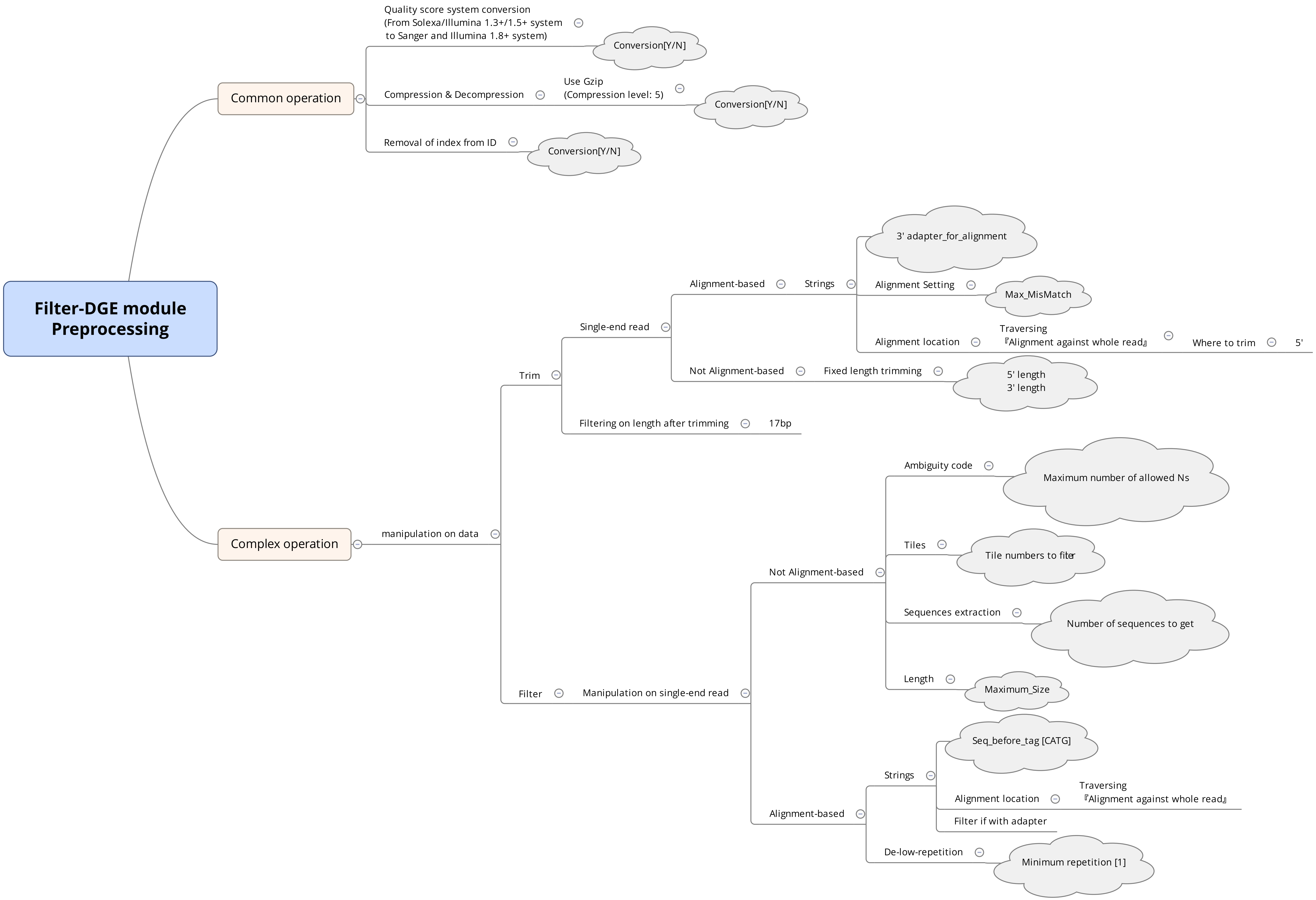
Filter-DGE module Preprocessing
Common operation
Quality score system conversion (From Solexa/Illumina 1.3+/1.5+ system to Sanger and Illumina 1.8+ system)
Conversion[Y/N]
Compression & Decompression
Use Gzip (Compression level: 5)
Conversion[Y/N]
Removal of index from ID
Conversion[Y/N]
Complex operation
manipulation on data
Trim
Single-end read
Alignment-based
Strings
3' adapter_for_alignment
Alignment Setting
Max_MisMatch
Alignment location
Traversing 『Alignment against whole read』
Where to trim
5'
Not Alignment-based
Fixed length trimming
5' length
3' length
Filtering on length after trimming
17bp
Filter
Manipulation on single-end read
Not Alignment-based
Ambiguity code
Maximum number of allowed Ns
Tiles
Tile numbers to filter
Sequences extraction
Number of sequences to get
Length
Maximum_Size
Alignment-based
Strings
Seq_before_tag [CATG]
Alignment location
Traversing 『Alignment against whole read』
Filter if with adapter
De-low-repetition
Minimum repetition [1]

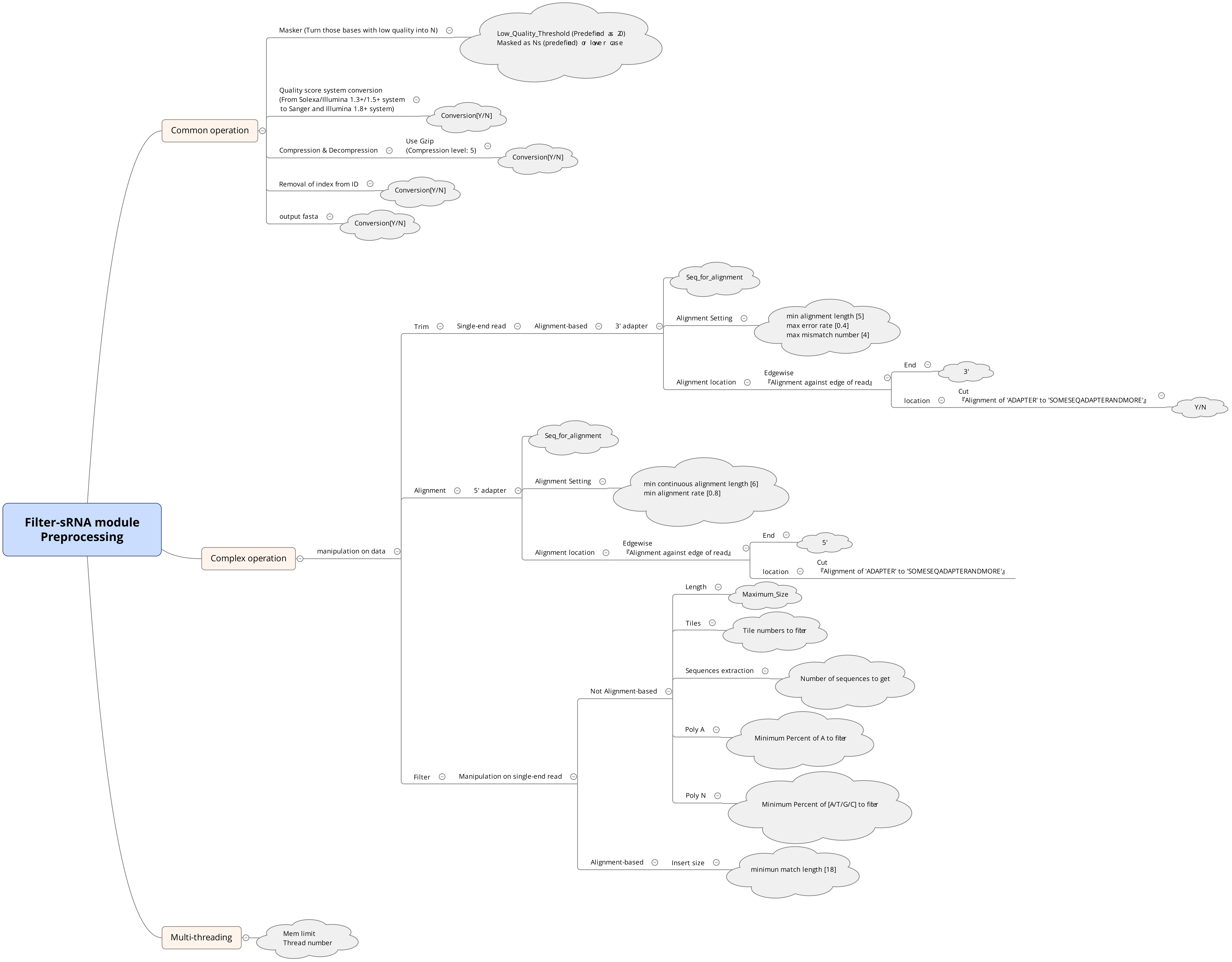
Filter-sRNA module
Preprocessing
Common operation
Masker (Turn those bases with low quality into N)
Low_Quality_Threshold (Predefined as 20)
Masked as Ns (predefined) or lower case
Quality score system conversion
(From Solexa/Illumina 1.3+/1.5+ system
to Sanger and Illumina 1.8+ system)
Conversion[Y/N]
Compression & Decompression
Use Gzip
(Compression level: 5)
Conversion[Y/N]
Removal of index from ID
Conversion[Y/N]
output fasta
Conversion[Y/N]
Complex operation
manipulation on data
Trim
Single-end read
Alignment-based
3' adapter
Seq_for_alignment
Alignment Setting
min alignment length [5]
max error rate [0.4]
max mismatch number [4]
Alignment location
Edgewise
『Alignment against edge of read』
End
3'
location
Cut
『Alignment of 'ADAPTER' to 'SOMESEQADAPTERANDMORE'』
Y/N
Alignment
5' adapter
Seq_for_alignment
Alignment Setting
min continuous alignment length [6]
min alignment rate [0.8]
Alignment location
Edgewise
『Alignment against edge of read』
End
5'
location
Cut
『Alignment of 'ADAPTER' to 'SOMESEQADAPTERANDMORE'』
Filter
Manipulation on single-end read
Not Alignment-based
Length
Maximum_Size
Tiles
Tile numbers to filter
Sequences extraction
Number of sequences to get
Poly A
Minimum Percent of A to filter
Poly N
Minimum Percent of [A/T/G/C] to filter
Alignment-based
Insert size
minimun match length [18]
Multi-threading
Mem limit
Thread number

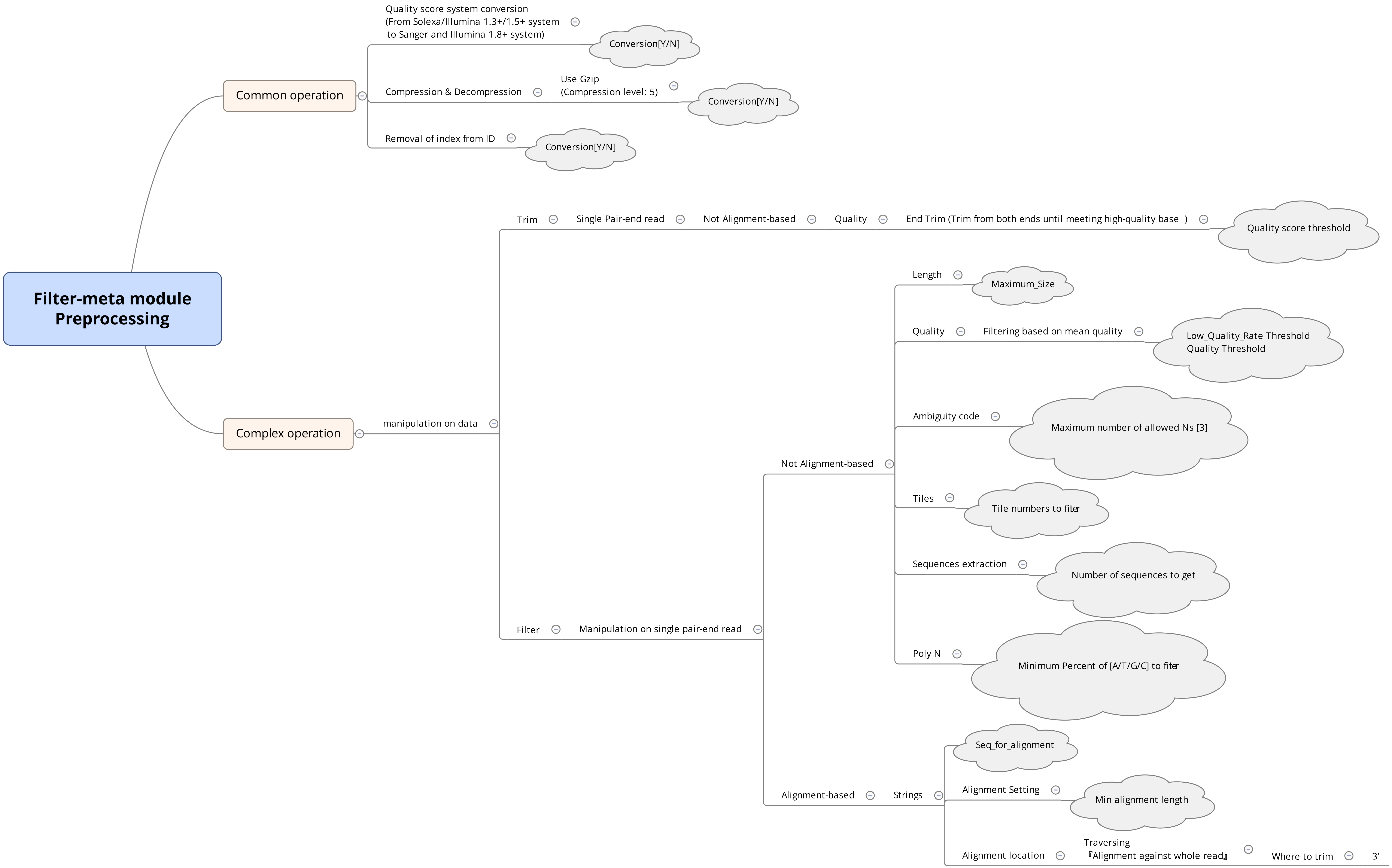
Filter-meta module
Preprocessing
Common operation
Quality score system conversion
(From Solexa/Illumina 1.3+/1.5+ system
to Sanger and Illumina 1.8+ system)
Conversion[Y/N]
Compression & Decompression
Use Gzip
(Compression level: 5)
Conversion[Y/N]
Removal of index from ID
Conversion[Y/N]
Complex operation
manipulation on data
Trim
Single Pair-end read
Not Alignment-based
Quality
End Trim (Trim from both ends until meeting high-quality base)
Quality score threshold
Filter
Manipulation on single pair-end read
Not Alignment-based
Length
Maximum_Size
Quality
Filtering based on mean quality
Low_Quality_Rate Threshold
Quality Threshold
Ambiguity code
Maximum number of allowed Ns [3]
Tiles
Tile numbers to filter
Sequences extraction
Number of sequences to get
Poly N
Minimum Percent of [A/T/G/C] to filter
Alignment-based
Strings
Seq_for_alignment
Alignment Setting
Min alignment length
Alignment location
Traversing
『Alignment against whole read』
Where to trim
3'
