# Supplementary material for: SOAPnuke: a MapReduce acceleration-supported software for integrated quality control and preprocessing of high-throughput sequencing data
Source: Gigascience. 2017 Dec 4;7(1):gix120. doi: 10.1093/gigascience/gix120 (PMC5788068; doi:10.1093/gigascience/gix120)
Supplement: Supplement materials [file gix120_supp.zip › SM4.docx]

Supplementary Material 4: Details of processing performance test on SOAPnuke and other tools.

Data for testing:

| PE1 download link | PE1 MD5 | PE2 download link | PE2 MD5 |
| --- | --- | --- | --- |
| ftp://ftp-trace.ncbi.nih.gov/giab/ftp/data/NA12878/NIST_NA12878_HG001_HiSeq_300x/140313_D00360_0015_BH9258ADXX/Project_RM8398/Sample_U5b/U5b_AGTTCC_L001_R1_001.fastq.gz | 1029c1c0493683ff1a1172c0e909780f | ftp://ftp-trace.ncbi.nih.gov/giab/ftp/data/NA12878/NIST_NA12878_HG001_HiSeq_300x/140313_D00360_0015_BH9258ADXX/Project_RM8398/Sample_U5b/U5b_AGTTCC_L001_R2_001.fastq.gz | b14954a36399cbe9f4963d75a63febb3 |
| ftp://ftp-trace.ncbi.nih.gov/giab/ftp/data/NA12878/NIST_NA12878_HG001_HiSeq_300x/140313_D00360_0015_BH9258ADXX/Project_RM8398/Sample_U5b/U5b_AGTTCC_L001_R1_002.fastq.gz | 74ea4dd6accf232c825d9be1b4cab537 | ftp://ftp-trace.ncbi.nih.gov/giab/ftp/data/NA12878/NIST_NA12878_HG001_HiSeq_300x/140313_D00360_0015_BH9258ADXX/Project_RM8398/Sample_U5b/U5b_AGTTCC_L001_R2_002.fastq.gz | a9de8c3bea52d36be47c52eae7491529 |
| ftp://ftp-trace.ncbi.nih.gov/giab/ftp/data/NA12878/NIST_NA12878_HG001_HiSeq_300x/140313_D00360_0015_BH9258ADXX/Project_RM8398/Sample_U5b/U5b_AGTTCC_L001_R1_003.fastq.gz | 36e26023bec8811de3ff16a549a562ee | ftp://ftp-trace.ncbi.nih.gov/giab/ftp/data/NA12878/NIST_NA12878_HG001_HiSeq_300x/140313_D00360_0015_BH9258ADXX/Project_RM8398/Sample_U5b/U5b_AGTTCC_L001_R2_003.fastq.gz | afd9832a9fbbd2b0d4222e5f52ea2ab2 |
| ftp://ftp-trace.ncbi.nih.gov/giab/ftp/data/NA12878/NIST_NA12878_HG001_HiSeq_300x/140313_D00360_0015_BH9258ADXX/Project_RM8398/Sample_U5b/U5b_AGTTCC_L001_R1_004.fastq.gz | 679de5cf8bd2fd7174ca314f54f4b13a | ftp://ftp-trace.ncbi.nih.gov/giab/ftp/data/NA12878/NIST_NA12878_HG001_HiSeq_300x/140313_D00360_0015_BH9258ADXX/Project_RM8398/Sample_U5b/U5b_AGTTCC_L001_R2_004.fastq.gz | 610b450d8d91102bf335df72c4ac6362 |
| ftp://ftp-trace.ncbi.nih.gov/giab/ftp/data/NA12878/NIST_NA12878_HG001_HiSeq_300x/140313_D00360_0015_BH9258ADXX/Project_RM8398/Sample_U5b/U5b_AGTTCC_L001_R1_005.fastq.gz | 03ec7f8d3059e47ff5e36121550302f4 | ftp://ftp-trace.ncbi.nih.gov/giab/ftp/data/NA12878/NIST_NA12878_HG001_HiSeq_300x/140313_D00360_0015_BH9258ADXX/Project_RM8398/Sample_U5b/U5b_AGTTCC_L001_R2_005.fastq.gz | cc1f653754078a3a9ec3f2a5fbcd7109 |
| ftp://ftp-trace.ncbi.nih.gov/giab/ftp/data/NA12878/NIST_NA12878_HG001_HiSeq_300x/140313_D00360_0015_BH9258ADXX/Project_RM8398/Sample_U5b/U5b_AGTTCC_L001_R1_006.fastq.gz | 4c8d13fb300fdf95ab41565449261036 | ftp://ftp-trace.ncbi.nih.gov/giab/ftp/data/NA12878/NIST_NA12878_HG001_HiSeq_300x/140313_D00360_0015_BH9258ADXX/Project_RM8398/Sample_U5b/U5b_AGTTCC_L001_R2_006.fastq.gz | 01d5df388d1cba254d49dbf7d6c43ae2 |
| ftp://ftp-trace.ncbi.nih.gov/giab/ftp/data/NA12878/NIST_NA12878_HG001_HiSeq_300x/140313_D00360_0015_BH9258ADXX/Project_RM8398/Sample_U5b/U5b_AGTTCC_L001_R1_007.fastq.gz | 31583503a60d2c80ae5781b43e2d1aa1 | ftp://ftp-trace.ncbi.nih.gov/giab/ftp/data/NA12878/NIST_NA12878_HG001_HiSeq_300x/140313_D00360_0015_BH9258ADXX/Project_RM8398/Sample_U5b/U5b_AGTTCC_L001_R2_007.fastq.gz | 6148c26a52ed4b5f16994ca2d76fa574 |
| ftp://ftp-trace.ncbi.nih.gov/giab/ftp/data/NA12878/NIST_NA12878_HG001_HiSeq_300x/140313_D00360_0015_BH9258ADXX/Project_RM8398/Sample_U5b/U5b_AGTTCC_L002_R1_001.fastq.gz | 6eaf0e042d9eb4f3fc76083544487631 | ftp://ftp-trace.ncbi.nih.gov/giab/ftp/data/NA12878/NIST_NA12878_HG001_HiSeq_300x/140313_D00360_0015_BH9258ADXX/Project_RM8398/Sample_U5b/U5b_AGTTCC_L002_R2_001.fastq.gz | 1dedbfabb827a2b1ea3883b1c6791984 |
| ftp://ftp-trace.ncbi.nih.gov/giab/ftp/data/NA12878/NIST_NA12878_HG001_HiSeq_300x/140313_D00360_0015_BH9258ADXX/Project_RM8398/Sample_U5b/U5b_AGTTCC_L002_R1_002.fastq.gz | e623b56d6a96e8078076dd72aa1867cc | ftp://ftp-trace.ncbi.nih.gov/giab/ftp/data/NA12878/NIST_NA12878_HG001_HiSeq_300x/140313_D00360_0015_BH9258ADXX/Project_RM8398/Sample_U5b/U5b_AGTTCC_L002_R2_002.fastq.gz | f01c9c9e8bc784ac4dbf4e167a598d66 |
| ftp://ftp-trace.ncbi.nih.gov/giab/ftp/data/NA12878/NIST_NA12878_HG001_HiSeq_300x/140313_D00360_0015_BH9258ADXX/Project_RM8398/Sample_U5b/U5b_AGTTCC_L002_R1_003.fastq.gz | 77dd39a89e2c14cd8f2589e6d1494e12 | ftp://ftp-trace.ncbi.nih.gov/giab/ftp/data/NA12878/NIST_NA12878_HG001_HiSeq_300x/140313_D00360_0015_BH9258ADXX/Project_RM8398/Sample_U5b/U5b_AGTTCC_L002_R2_003.fastq.gz | da18d6b992b18be09794bab576f26138 |
| ftp://ftp-trace.ncbi.nih.gov/giab/ftp/data/NA12878/NIST_NA12878_HG001_HiSeq_300x/140313_D00360_0015_BH9258ADXX/Project_RM8398/Sample_U5b/U5b_AGTTCC_L002_R1_004.fastq.gz | 86feaa9bb658ee99a97caff4cb5e1f7f | ftp://ftp-trace.ncbi.nih.gov/giab/ftp/data/NA12878/NIST_NA12878_HG001_HiSeq_300x/140313_D00360_0015_BH9258ADXX/Project_RM8398/Sample_U5b/U5b_AGTTCC_L002_R2_004.fastq.gz | f803dde910f995d8c77d81b78ca60fac |
| ftp://ftp-trace.ncbi.nih.gov/giab/ftp/data/NA12878/NIST_NA12878_HG001_HiSeq_300x/140313_D00360_0015_BH9258ADXX/Project_RM8398/Sample_U5b/U5b_AGTTCC_L002_R1_005.fastq.gz | f753b7c8c021aa9ac58c435449f8dab4 | ftp://ftp-trace.ncbi.nih.gov/giab/ftp/data/NA12878/NIST_NA12878_HG001_HiSeq_300x/140313_D00360_0015_BH9258ADXX/Project_RM8398/Sample_U5b/U5b_AGTTCC_L002_R2_005.fastq.gz | a92713935a7337665fa38513eabc046f |
| ftp://ftp-trace.ncbi.nih.gov/giab/ftp/data/NA12878/NIST_NA12878_HG001_HiSeq_300x/140313_D00360_0015_BH9258ADXX/Project_RM8398/Sample_U5b/U5b_AGTTCC_L002_R1_006.fastq.gz | b1dfb82c8058fae7140410142b521eb5 | ftp://ftp-trace.ncbi.nih.gov/giab/ftp/data/NA12878/NIST_NA12878_HG001_HiSeq_300x/140313_D00360_0015_BH9258ADXX/Project_RM8398/Sample_U5b/U5b_AGTTCC_L002_R2_006.fastq.gz | 4afdd0c36df735db7620de073f278500 |
| ftp://ftp-trace.ncbi.nih.gov/giab/ftp/data/NA12878/NIST_NA12878_HG001_HiSeq_300x/140313_D00360_0015_BH9258ADXX/Project_RM8398/Sample_U5b/U5b_AGTTCC_L002_R1_007.fastq.gz | 0488149b9e46ce17e45885bc1f716bc0 | ftp://ftp-trace.ncbi.nih.gov/giab/ftp/data/NA12878/NIST_NA12878_HG001_HiSeq_300x/140313_D00360_0015_BH9258ADXX/Project_RM8398/Sample_U5b/U5b_AGTTCC_L002_R2_007.fastq.gz | dc217916d1c230f08f359e481645e5c6 |
| ftp://ftp-trace.ncbi.nih.gov/giab/ftp/data/NA12878/NIST_NA12878_HG001_HiSeq_300x/140407_D00360_0016_AH948VADXX/Project_RM8398/Sample_U5b/U5b_AGTTCC_L001_R1_001.fastq.gz | 149a95e26723652040381779d29d3319 | ftp://ftp-trace.ncbi.nih.gov/giab/ftp/data/NA12878/NIST_NA12878_HG001_HiSeq_300x/140407_D00360_0016_AH948VADXX/Project_RM8398/Sample_U5b/U5b_AGTTCC_L001_R2_001.fastq.gz | 77952cdbe67c4221fe336fcc8a31e115 |
| ftp://ftp-trace.ncbi.nih.gov/giab/ftp/data/NA12878/NIST_NA12878_HG001_HiSeq_300x/140407_D00360_0016_AH948VADXX/Project_RM8398/Sample_U5b/U5b_AGTTCC_L001_R1_002.fastq.gz | ae87db25045aff686f6a12491dbde095 | ftp://ftp-trace.ncbi.nih.gov/giab/ftp/data/NA12878/NIST_NA12878_HG001_HiSeq_300x/140407_D00360_0016_AH948VADXX/Project_RM8398/Sample_U5b/U5b_AGTTCC_L001_R2_002.fastq.gz | c401073324a1659482bea5b3d409f2b8 |
| ftp://ftp-trace.ncbi.nih.gov/giab/ftp/data/NA12878/NIST_NA12878_HG001_HiSeq_300x/140407_D00360_0016_AH948VADXX/Project_RM8398/Sample_U5b/U5b_AGTTCC_L001_R1_003.fastq.gz | df7adc860bea845425c89af6f9acd4e5 | ftp://ftp-trace.ncbi.nih.gov/giab/ftp/data/NA12878/NIST_NA12878_HG001_HiSeq_300x/140407_D00360_0016_AH948VADXX/Project_RM8398/Sample_U5b/U5b_AGTTCC_L001_R2_003.fastq.gz | 7e721da7d77c058868faece7f3c53283 |
| ftp://ftp-trace.ncbi.nih.gov/giab/ftp/data/NA12878/NIST_NA12878_HG001_HiSeq_300x/140407_D00360_0016_AH948VADXX/Project_RM8398/Sample_U5b/U5b_AGTTCC_L001_R1_004.fastq.gz | 77713ea1452efe79a30e410e6c18df3b | ftp://ftp-trace.ncbi.nih.gov/giab/ftp/data/NA12878/NIST_NA12878_HG001_HiSeq_300x/140407_D00360_0016_AH948VADXX/Project_RM8398/Sample_U5b/U5b_AGTTCC_L001_R2_004.fastq.gz | b5e37fa89c216431b0470ef61d91f022 |
| ftp://ftp-trace.ncbi.nih.gov/giab/ftp/data/NA12878/NIST_NA12878_HG001_HiSeq_300x/140407_D00360_0016_AH948VADXX/Project_RM8398/Sample_U5b/U5b_AGTTCC_L001_R1_005.fastq.gz | 3ca9ef3cc6ca773c3512db2daef5422a | ftp://ftp-trace.ncbi.nih.gov/giab/ftp/data/NA12878/NIST_NA12878_HG001_HiSeq_300x/140407_D00360_0016_AH948VADXX/Project_RM8398/Sample_U5b/U5b_AGTTCC_L001_R2_005.fastq.gz | 919c0101ac4634fd9dc333b38c509083 |
| ftp://ftp-trace.ncbi.nih.gov/giab/ftp/data/NA12878/NIST_NA12878_HG001_HiSeq_300x/140407_D00360_0016_AH948VADXX/Project_RM8398/Sample_U5b/U5b_AGTTCC_L002_R1_001.fastq.gz | ba0226218933b6db1f0bd375453f3278 | ftp://ftp-trace.ncbi.nih.gov/giab/ftp/data/NA12878/NIST_NA12878_HG001_HiSeq_300x/140407_D00360_0016_AH948VADXX/Project_RM8398/Sample_U5b/U5b_AGTTCC_L002_R2_001.fastq.gz | 7665e623b853efd434abc8da998adc29 |
| ftp://ftp-trace.ncbi.nih.gov/giab/ftp/data/NA12878/NIST_NA12878_HG001_HiSeq_300x/140407_D00360_0016_AH948VADXX/Project_RM8398/Sample_U5b/U5b_AGTTCC_L002_R1_002.fastq.gz | 42defd8674c787654676be6de7069dd6 | ftp://ftp-trace.ncbi.nih.gov/giab/ftp/data/NA12878/NIST_NA12878_HG001_HiSeq_300x/140407_D00360_0016_AH948VADXX/Project_RM8398/Sample_U5b/U5b_AGTTCC_L002_R2_002.fastq.gz | a08f62b41eea54d81027fa25b0016df6 |
| ftp://ftp-trace.ncbi.nih.gov/giab/ftp/data/NA12878/NIST_NA12878_HG001_HiSeq_300x/140407_D00360_0016_AH948VADXX/Project_RM8398/Sample_U5b/U5b_AGTTCC_L002_R1_003.fastq.gz | c3c3e02ac40eb684d454e744ca9e4775 | ftp://ftp-trace.ncbi.nih.gov/giab/ftp/data/NA12878/NIST_NA12878_HG001_HiSeq_300x/140407_D00360_0016_AH948VADXX/Project_RM8398/Sample_U5b/U5b_AGTTCC_L002_R2_003.fastq.gz | 3003ef8d1613a633bc1adcbc412363bb |
| ftp://ftp-trace.ncbi.nih.gov/giab/ftp/data/NA12878/NIST_NA12878_HG001_HiSeq_300x/140407_D00360_0016_AH948VADXX/Project_RM8398/Sample_U5b/U5b_AGTTCC_L002_R1_004.fastq.gz | 76feb088c2b85144dd7ab3afd25f04ce | ftp://ftp-trace.ncbi.nih.gov/giab/ftp/data/NA12878/NIST_NA12878_HG001_HiSeq_300x/140407_D00360_0016_AH948VADXX/Project_RM8398/Sample_U5b/U5b_AGTTCC_L002_R2_004.fastq.gz | ee0894c5c53dd37e4482a7cad16b0ad7 |
| ftp://ftp-trace.ncbi.nih.gov/giab/ftp/data/NA12878/NIST_NA12878_HG001_HiSeq_300x/140407_D00360_0016_AH948VADXX/Project_RM8398/Sample_U5b/U5b_AGTTCC_L002_R1_005.fastq.gz | 149a2fd050a9deb2d4abe868862f6029 | ftp://ftp-trace.ncbi.nih.gov/giab/ftp/data/NA12878/NIST_NA12878_HG001_HiSeq_300x/140407_D00360_0016_AH948VADXX/Project_RM8398/Sample_U5b/U5b_AGTTCC_L002_R2_005.fastq.gz | 6afea41bfa1b051a7c532e073d7c873c |
| ftp://ftp-trace.ncbi.nih.gov/giab/ftp/data/NA12878/NIST_NA12878_HG001_HiSeq_300x/140407_D00360_0017_BH947YADXX/Project_RM8398/Sample_U0a/U0a_CGATGT_L001_R1_001.fastq.gz | f553a37be98ecfd6b3be6b798f8100cb | ftp://ftp-trace.ncbi.nih.gov/giab/ftp/data/NA12878/NIST_NA12878_HG001_HiSeq_300x/140407_D00360_0017_BH947YADXX/Project_RM8398/Sample_U0a/U0a_CGATGT_L001_R2_001.fastq.gz | 47d4d7e96c9c0f5044ed43e8981cee49 |
| ftp://ftp-trace.ncbi.nih.gov/giab/ftp/data/NA12878/NIST_NA12878_HG001_HiSeq_300x/140407_D00360_0017_BH947YADXX/Project_RM8398/Sample_U0a/U0a_CGATGT_L001_R1_002.fastq.gz | de93c1378cbe7853cdacd4ad6558638f | ftp://ftp-trace.ncbi.nih.gov/giab/ftp/data/NA12878/NIST_NA12878_HG001_HiSeq_300x/140407_D00360_0017_BH947YADXX/Project_RM8398/Sample_U0a/U0a_CGATGT_L001_R2_002.fastq.gz | 082f3644c32fe77d7e0ac1ddadf13d0b |
| ftp://ftp-trace.ncbi.nih.gov/giab/ftp/data/NA12878/NIST_NA12878_HG001_HiSeq_300x/140407_D00360_0017_BH947YADXX/Project_RM8398/Sample_U0a/U0a_CGATGT_L001_R1_003.fastq.gz | 2be64ea2cbd8107d5e534b09517dcce5 | ftp://ftp-trace.ncbi.nih.gov/giab/ftp/data/NA12878/NIST_NA12878_HG001_HiSeq_300x/140407_D00360_0017_BH947YADXX/Project_RM8398/Sample_U0a/U0a_CGATGT_L001_R2_003.fastq.gz | c2fc1459022fabaf6765431859840cdf |
| ftp://ftp-trace.ncbi.nih.gov/giab/ftp/data/NA12878/NIST_NA12878_HG001_HiSeq_300x/140407_D00360_0017_BH947YADXX/Project_RM8398/Sample_U0a/U0a_CGATGT_L001_R1_004.fastq.gz | 6250cc6a9f4000bfe307256b55d4d802 | ftp://ftp-trace.ncbi.nih.gov/giab/ftp/data/NA12878/NIST_NA12878_HG001_HiSeq_300x/140407_D00360_0017_BH947YADXX/Project_RM8398/Sample_U0a/U0a_CGATGT_L001_R2_004.fastq.gz | e8ff09b0b6a89ecc1f37912425d0ac1a |
| ftp://ftp-trace.ncbi.nih.gov/giab/ftp/data/NA12878/NIST_NA12878_HG001_HiSeq_300x/140407_D00360_0017_BH947YADXX/Project_RM8398/Sample_U0a/U0a_CGATGT_L002_R1_001.fastq.gz | a8c120dbd58507971d9e3c3e1df76293 | ftp://ftp-trace.ncbi.nih.gov/giab/ftp/data/NA12878/NIST_NA12878_HG001_HiSeq_300x/140407_D00360_0017_BH947YADXX/Project_RM8398/Sample_U0a/U0a_CGATGT_L002_R2_001.fastq.gz | a2efd4d179eedba82f04adf612968b5b |
| ftp://ftp-trace.ncbi.nih.gov/giab/ftp/data/NA12878/NIST_NA12878_HG001_HiSeq_300x/140407_D00360_0017_BH947YADXX/Project_RM8398/Sample_U0a/U0a_CGATGT_L002_R1_002.fastq.gz | e7ebcfd669f81d11eee5f33c12ad6d86 | ftp://ftp-trace.ncbi.nih.gov/giab/ftp/data/NA12878/NIST_NA12878_HG001_HiSeq_300x/140407_D00360_0017_BH947YADXX/Project_RM8398/Sample_U0a/U0a_CGATGT_L002_R2_002.fastq.gz | 2f12e19c04a53dd4f0cf4d8949b1770b |
| ftp://ftp-trace.ncbi.nih.gov/giab/ftp/data/NA12878/NIST_NA12878_HG001_HiSeq_300x/140407_D00360_0017_BH947YADXX/Project_RM8398/Sample_U0a/U0a_CGATGT_L002_R1_003.fastq.gz | f49059e0c70cb8be737127d692b7870e | ftp://ftp-trace.ncbi.nih.gov/giab/ftp/data/NA12878/NIST_NA12878_HG001_HiSeq_300x/140407_D00360_0017_BH947YADXX/Project_RM8398/Sample_U0a/U0a_CGATGT_L002_R2_003.fastq.gz | 03da880565859820e67c52ab64b3dd48 |
| ftp://ftp-trace.ncbi.nih.gov/giab/ftp/data/NA12878/NIST_NA12878_HG001_HiSeq_300x/140407_D00360_0017_BH947YADXX/Project_RM8398/Sample_U0a/U0a_CGATGT_L002_R1_004.fastq.gz | d4aa024163767aebcd1e282557d863ee | ftp://ftp-trace.ncbi.nih.gov/giab/ftp/data/NA12878/NIST_NA12878_HG001_HiSeq_300x/140407_D00360_0017_BH947YADXX/Project_RM8398/Sample_U0a/U0a_CGATGT_L002_R2_004.fastq.gz | 7400226108329adeaec4b5d0b54a47a9 |
| ftp://ftp-trace.ncbi.nih.gov/giab/ftp/data/NA12878/NIST_NA12878_HG001_HiSeq_300x/140407_D00360_0017_BH947YADXX/Project_RM8398/Sample_U0b/U0b_TGACCA_L001_R1_001.fastq.gz | bbd7e61417d45f5d12e0b27e44077c34 | ftp://ftp-trace.ncbi.nih.gov/giab/ftp/data/NA12878/NIST_NA12878_HG001_HiSeq_300x/140407_D00360_0017_BH947YADXX/Project_RM8398/Sample_U0b/U0b_TGACCA_L001_R2_001.fastq.gz | 00eeefafb6dcd25560de8112e7d45914 |
| ftp://ftp-trace.ncbi.nih.gov/giab/ftp/data/NA12878/NIST_NA12878_HG001_HiSeq_300x/140407_D00360_0017_BH947YADXX/Project_RM8398/Sample_U0b/U0b_TGACCA_L001_R1_002.fastq.gz | d1f344421100d9d29c248c763ef32e40 | ftp://ftp-trace.ncbi.nih.gov/giab/ftp/data/NA12878/NIST_NA12878_HG001_HiSeq_300x/140407_D00360_0017_BH947YADXX/Project_RM8398/Sample_U0b/U0b_TGACCA_L001_R2_002.fastq.gz | d919dfeb72085a28e9452d890c4f781d |
| ftp://ftp-trace.ncbi.nih.gov/giab/ftp/data/NA12878/NIST_NA12878_HG001_HiSeq_300x/140407_D00360_0017_BH947YADXX/Project_RM8398/Sample_U0b/U0b_TGACCA_L001_R1_003.fastq.gz | 0d974bcf70bc21302a4df6b23c6f1c9a | ftp://ftp-trace.ncbi.nih.gov/giab/ftp/data/NA12878/NIST_NA12878_HG001_HiSeq_300x/140407_D00360_0017_BH947YADXX/Project_RM8398/Sample_U0b/U0b_TGACCA_L001_R2_003.fastq.gz | 59d7cad7e428ae905e74bf7d957ef45d |
| ftp://ftp-trace.ncbi.nih.gov/giab/ftp/data/NA12878/NIST_NA12878_HG001_HiSeq_300x/140407_D00360_0017_BH947YADXX/Project_RM8398/Sample_U0b/U0b_TGACCA_L001_R1_004.fastq.gz | b1d7a80369ddc822c4aba34f996f2afb | ftp://ftp-trace.ncbi.nih.gov/giab/ftp/data/NA12878/NIST_NA12878_HG001_HiSeq_300x/140407_D00360_0017_BH947YADXX/Project_RM8398/Sample_U0b/U0b_TGACCA_L001_R2_004.fastq.gz | f0f6d00a8239ee80df56f6d6a86de233 |
| ftp://ftp-trace.ncbi.nih.gov/giab/ftp/data/NA12878/NIST_NA12878_HG001_HiSeq_300x/140407_D00360_0017_BH947YADXX/Project_RM8398/Sample_U0b/U0b_TGACCA_L001_R1_005.fastq.gz | 37e8ffbcf4d1c610419d861221772ba0 | ftp://ftp-trace.ncbi.nih.gov/giab/ftp/data/NA12878/NIST_NA12878_HG001_HiSeq_300x/140407_D00360_0017_BH947YADXX/Project_RM8398/Sample_U0b/U0b_TGACCA_L001_R2_005.fastq.gz | bf54cde3bd471c407f0d543ddfaf3615 |
| ftp://ftp-trace.ncbi.nih.gov/giab/ftp/data/NA12878/NIST_NA12878_HG001_HiSeq_300x/140407_D00360_0017_BH947YADXX/Project_RM8398/Sample_U0b/U0b_TGACCA_L002_R1_001.fastq.gz | 12dfed9a08481e38d6e64226e9ecc78b | ftp://ftp-trace.ncbi.nih.gov/giab/ftp/data/NA12878/NIST_NA12878_HG001_HiSeq_300x/140407_D00360_0017_BH947YADXX/Project_RM8398/Sample_U0b/U0b_TGACCA_L002_R2_001.fastq.gz | d272a12fdfcdbb6e519fc711e13c57a7 |
| ftp://ftp-trace.ncbi.nih.gov/giab/ftp/data/NA12878/NIST_NA12878_HG001_HiSeq_300x/140407_D00360_0017_BH947YADXX/Project_RM8398/Sample_U0b/U0b_TGACCA_L002_R1_002.fastq.gz | 552dd958cc305043af035bbb48c18ac5 | ftp://ftp-trace.ncbi.nih.gov/giab/ftp/data/NA12878/NIST_NA12878_HG001_HiSeq_300x/140407_D00360_0017_BH947YADXX/Project_RM8398/Sample_U0b/U0b_TGACCA_L002_R2_002.fastq.gz | baabb64cccb6df0bd67c268aeb94f327 |
| ftp://ftp-trace.ncbi.nih.gov/giab/ftp/data/NA12878/NIST_NA12878_HG001_HiSeq_300x/140407_D00360_0017_BH947YADXX/Project_RM8398/Sample_U0b/U0b_TGACCA_L002_R1_003.fastq.gz | 3bb0a9f4643a22221184c1741f9d3ba6 | ftp://ftp-trace.ncbi.nih.gov/giab/ftp/data/NA12878/NIST_NA12878_HG001_HiSeq_300x/140407_D00360_0017_BH947YADXX/Project_RM8398/Sample_U0b/U0b_TGACCA_L002_R2_003.fastq.gz | 792d57436a95aa3a30929d56942ef6c6 |
| ftp://ftp-trace.ncbi.nih.gov/giab/ftp/data/NA12878/NIST_NA12878_HG001_HiSeq_300x/140407_D00360_0017_BH947YADXX/Project_RM8398/Sample_U0b/U0b_TGACCA_L002_R1_004.fastq.gz | 1721ec1ad4d0015658e448860dde9ea4 | ftp://ftp-trace.ncbi.nih.gov/giab/ftp/data/NA12878/NIST_NA12878_HG001_HiSeq_300x/140407_D00360_0017_BH947YADXX/Project_RM8398/Sample_U0b/U0b_TGACCA_L002_R2_004.fastq.gz | af10de071fd3c0ea1f9b57ee0265ef99 |
| ftp://ftp-trace.ncbi.nih.gov/giab/ftp/data/NA12878/NIST_NA12878_HG001_HiSeq_300x/140407_D00360_0017_BH947YADXX/Project_RM8398/Sample_U0b/U0b_TGACCA_L002_R1_005.fastq.gz | c430f74c3d18cd21cc60c2765f19887c | ftp://ftp-trace.ncbi.nih.gov/giab/ftp/data/NA12878/NIST_NA12878_HG001_HiSeq_300x/140407_D00360_0017_BH947YADXX/Project_RM8398/Sample_U0b/U0b_TGACCA_L002_R2_005.fastq.gz | 078eb54da98a3129ad7bcf739dc02886 |
| ftp://ftp-trace.ncbi.nih.gov/giab/ftp/data/NA12878/NIST_NA12878_HG001_HiSeq_300x/140407_D00360_0017_BH947YADXX/Project_RM8398/Sample_U0c/U0c_CAGATC_L001_R1_001.fastq.gz | cebfac64c3de28c93129d92d3234dafd | ftp://ftp-trace.ncbi.nih.gov/giab/ftp/data/NA12878/NIST_NA12878_HG001_HiSeq_300x/140407_D00360_0017_BH947YADXX/Project_RM8398/Sample_U0c/U0c_CAGATC_L001_R2_001.fastq.gz | dfc12982ee2493f8d511f0f1f348751e |
| ftp://ftp-trace.ncbi.nih.gov/giab/ftp/data/NA12878/NIST_NA12878_HG001_HiSeq_300x/140407_D00360_0017_BH947YADXX/Project_RM8398/Sample_U0c/U0c_CAGATC_L001_R1_002.fastq.gz | 9554c7df8d8659cebd2679f7e61838a5 | ftp://ftp-trace.ncbi.nih.gov/giab/ftp/data/NA12878/NIST_NA12878_HG001_HiSeq_300x/140407_D00360_0017_BH947YADXX/Project_RM8398/Sample_U0c/U0c_CAGATC_L001_R2_002.fastq.gz | 9bda8de751279dda389705f8cc242b0b |
| ftp://ftp-trace.ncbi.nih.gov/giab/ftp/data/NA12878/NIST_NA12878_HG001_HiSeq_300x/140407_D00360_0017_BH947YADXX/Project_RM8398/Sample_U0c/U0c_CAGATC_L001_R1_003.fastq.gz | 837ac7e80431681333c1a13d18b344ad | ftp://ftp-trace.ncbi.nih.gov/giab/ftp/data/NA12878/NIST_NA12878_HG001_HiSeq_300x/140407_D00360_0017_BH947YADXX/Project_RM8398/Sample_U0c/U0c_CAGATC_L001_R2_003.fastq.gz | b4290f7151f3cae796e813b7754f3d63 |
| ftp://ftp-trace.ncbi.nih.gov/giab/ftp/data/NA12878/NIST_NA12878_HG001_HiSeq_300x/140407_D00360_0017_BH947YADXX/Project_RM8398/Sample_U0c/U0c_CAGATC_L001_R1_004.fastq.gz | 0316424d2971ac6389c8db6bd05656f0 | ftp://ftp-trace.ncbi.nih.gov/giab/ftp/data/NA12878/NIST_NA12878_HG001_HiSeq_300x/140407_D00360_0017_BH947YADXX/Project_RM8398/Sample_U0c/U0c_CAGATC_L001_R2_004.fastq.gz | 0c4a0f02dbdc4053000d1ffa33768668 |
| ftp://ftp-trace.ncbi.nih.gov/giab/ftp/data/NA12878/NIST_NA12878_HG001_HiSeq_300x/140407_D00360_0017_BH947YADXX/Project_RM8398/Sample_U0c/U0c_CAGATC_L001_R1_005.fastq.gz | 8d20578f303f9eb45ba039cc25759a42 | ftp://ftp-trace.ncbi.nih.gov/giab/ftp/data/NA12878/NIST_NA12878_HG001_HiSeq_300x/140407_D00360_0017_BH947YADXX/Project_RM8398/Sample_U0c/U0c_CAGATC_L001_R2_005.fastq.gz | 9f778f1818b1bba6f9326941d0d8856b |
| ftp://ftp-trace.ncbi.nih.gov/giab/ftp/data/NA12878/NIST_NA12878_HG001_HiSeq_300x/140407_D00360_0017_BH947YADXX/Project_RM8398/Sample_U0c/U0c_CAGATC_L001_R1_006.fastq.gz | e958c87ee24538c52df8e50ff0664fd3 | ftp://ftp-trace.ncbi.nih.gov/giab/ftp/data/NA12878/NIST_NA12878_HG001_HiSeq_300x/140407_D00360_0017_BH947YADXX/Project_RM8398/Sample_U0c/U0c_CAGATC_L001_R2_006.fastq.gz | a789e748b776a67be162ec2759ebd155 |
| ftp://ftp-trace.ncbi.nih.gov/giab/ftp/data/NA12878/NIST_NA12878_HG001_HiSeq_300x/140407_D00360_0017_BH947YADXX/Project_RM8398/Sample_U0c/U0c_CAGATC_L002_R1_001.fastq.gz | 234af7749a084d5ea2f6a40d28d42d32 | ftp://ftp-trace.ncbi.nih.gov/giab/ftp/data/NA12878/NIST_NA12878_HG001_HiSeq_300x/140407_D00360_0017_BH947YADXX/Project_RM8398/Sample_U0c/U0c_CAGATC_L002_R2_001.fastq.gz | 2dda1aa4f4dd09c54a0ec03b367ff47e |
| ftp://ftp-trace.ncbi.nih.gov/giab/ftp/data/NA12878/NIST_NA12878_HG001_HiSeq_300x/140407_D00360_0017_BH947YADXX/Project_RM8398/Sample_U0c/U0c_CAGATC_L002_R1_002.fastq.gz | 28b494ce82baefdb49eb52bb20ecf88f | ftp://ftp-trace.ncbi.nih.gov/giab/ftp/data/NA12878/NIST_NA12878_HG001_HiSeq_300x/140407_D00360_0017_BH947YADXX/Project_RM8398/Sample_U0c/U0c_CAGATC_L002_R2_002.fastq.gz | 5b7e9c012da9604f250198be7c4747c8 |
| ftp://ftp-trace.ncbi.nih.gov/giab/ftp/data/NA12878/NIST_NA12878_HG001_HiSeq_300x/140407_D00360_0017_BH947YADXX/Project_RM8398/Sample_U0c/U0c_CAGATC_L002_R1_003.fastq.gz | f66c15afea5688442154d11094370ff5 | ftp://ftp-trace.ncbi.nih.gov/giab/ftp/data/NA12878/NIST_NA12878_HG001_HiSeq_300x/140407_D00360_0017_BH947YADXX/Project_RM8398/Sample_U0c/U0c_CAGATC_L002_R2_003.fastq.gz | 7f5aa75906018c9d868b71c890ede7b4 |
| ftp://ftp-trace.ncbi.nih.gov/giab/ftp/data/NA12878/NIST_NA12878_HG001_HiSeq_300x/140407_D00360_0017_BH947YADXX/Project_RM8398/Sample_U0c/U0c_CAGATC_L002_R1_004.fastq.gz | c641663e43730edd1dcca6d536e90112 | ftp://ftp-trace.ncbi.nih.gov/giab/ftp/data/NA12878/NIST_NA12878_HG001_HiSeq_300x/140407_D00360_0017_BH947YADXX/Project_RM8398/Sample_U0c/U0c_CAGATC_L002_R2_004.fastq.gz | c5f1f4b4fff34eb947003c329c0831a6 |
| ftp://ftp-trace.ncbi.nih.gov/giab/ftp/data/NA12878/NIST_NA12878_HG001_HiSeq_300x/140407_D00360_0017_BH947YADXX/Project_RM8398/Sample_U0c/U0c_CAGATC_L002_R1_005.fastq.gz | d4da66733896ce81dc19913b2b6e59de | ftp://ftp-trace.ncbi.nih.gov/giab/ftp/data/NA12878/NIST_NA12878_HG001_HiSeq_300x/140407_D00360_0017_BH947YADXX/Project_RM8398/Sample_U0c/U0c_CAGATC_L002_R2_005.fastq.gz | fd2cf275789fb00875778d522181a6ef |
| ftp://ftp-trace.ncbi.nih.gov/giab/ftp/data/NA12878/NIST_NA12878_HG001_HiSeq_300x/140407_D00360_0017_BH947YADXX/Project_RM8398/Sample_U0c/U0c_CAGATC_L002_R1_006.fastq.gz | d9f0dcfd8315c929054e705636dddcd9 | ftp://ftp-trace.ncbi.nih.gov/giab/ftp/data/NA12878/NIST_NA12878_HG001_HiSeq_300x/140407_D00360_0017_BH947YADXX/Project_RM8398/Sample_U0c/U0c_CAGATC_L002_R2_006.fastq.gz | 91c6436e60a25c78802a25b3992df8f8 |
| ftp://ftp-trace.ncbi.nih.gov/giab/ftp/data/NA12878/NIST_NA12878_HG001_HiSeq_300x/140407_D00360_0017_BH947YADXX/Project_RM8398/Sample_U5a/U5a_AGTCAA_L001_R1_001.fastq.gz | a38ee8e39e701b7eeba58046835499bf | ftp://ftp-trace.ncbi.nih.gov/giab/ftp/data/NA12878/NIST_NA12878_HG001_HiSeq_300x/140407_D00360_0017_BH947YADXX/Project_RM8398/Sample_U5a/U5a_AGTCAA_L001_R2_001.fastq.gz | b9fdc5b7ecb2a713556e78c0f44b4641 |
| ftp://ftp-trace.ncbi.nih.gov/giab/ftp/data/NA12878/NIST_NA12878_HG001_HiSeq_300x/140407_D00360_0017_BH947YADXX/Project_RM8398/Sample_U5a/U5a_AGTCAA_L001_R1_002.fastq.gz | 1ae015e993624b6d67be39ba7c96aa80 | ftp://ftp-trace.ncbi.nih.gov/giab/ftp/data/NA12878/NIST_NA12878_HG001_HiSeq_300x/140407_D00360_0017_BH947YADXX/Project_RM8398/Sample_U5a/U5a_AGTCAA_L001_R2_002.fastq.gz | 0b6348176640a7460bb13df549273108 |
| ftp://ftp-trace.ncbi.nih.gov/giab/ftp/data/NA12878/NIST_NA12878_HG001_HiSeq_300x/140407_D00360_0017_BH947YADXX/Project_RM8398/Sample_U5a/U5a_AGTCAA_L001_R1_003.fastq.gz | 79922aa93c20880577785afb83040fca | ftp://ftp-trace.ncbi.nih.gov/giab/ftp/data/NA12878/NIST_NA12878_HG001_HiSeq_300x/140407_D00360_0017_BH947YADXX/Project_RM8398/Sample_U5a/U5a_AGTCAA_L001_R2_003.fastq.gz | ee2ceb175038d4b8f7693c059ade6cb1 |
| ftp://ftp-trace.ncbi.nih.gov/giab/ftp/data/NA12878/NIST_NA12878_HG001_HiSeq_300x/140407_D00360_0017_BH947YADXX/Project_RM8398/Sample_U5a/U5a_AGTCAA_L001_R1_004.fastq.gz | bb637fab37e685869eaf2a4dd16b9673 | ftp://ftp-trace.ncbi.nih.gov/giab/ftp/data/NA12878/NIST_NA12878_HG001_HiSeq_300x/140407_D00360_0017_BH947YADXX/Project_RM8398/Sample_U5a/U5a_AGTCAA_L001_R2_004.fastq.gz | a40ad5e4f032b1704aa43a5b1e539a83 |
| ftp://ftp-trace.ncbi.nih.gov/giab/ftp/data/NA12878/NIST_NA12878_HG001_HiSeq_300x/140407_D00360_0017_BH947YADXX/Project_RM8398/Sample_U5a/U5a_AGTCAA_L001_R1_005.fastq.gz | 271b8f26b153297d910b0ca69327fc44 | ftp://ftp-trace.ncbi.nih.gov/giab/ftp/data/NA12878/NIST_NA12878_HG001_HiSeq_300x/140407_D00360_0017_BH947YADXX/Project_RM8398/Sample_U5a/U5a_AGTCAA_L001_R2_005.fastq.gz | 601d4999e7b4b7cd249dade94df94116 |
| ftp://ftp-trace.ncbi.nih.gov/giab/ftp/data/NA12878/NIST_NA12878_HG001_HiSeq_300x/140407_D00360_0017_BH947YADXX/Project_RM8398/Sample_U5a/U5a_AGTCAA_L001_R1_006.fastq.gz | e8c6bb409e212c8e651b0e2fbfab8b53 | ftp://ftp-trace.ncbi.nih.gov/giab/ftp/data/NA12878/NIST_NA12878_HG001_HiSeq_300x/140407_D00360_0017_BH947YADXX/Project_RM8398/Sample_U5a/U5a_AGTCAA_L001_R2_006.fastq.gz | 8b6300f635c8356a6ea525aa08557e2c |
| ftp://ftp-trace.ncbi.nih.gov/giab/ftp/data/NA12878/NIST_NA12878_HG001_HiSeq_300x/140407_D00360_0017_BH947YADXX/Project_RM8398/Sample_U5a/U5a_AGTCAA_L002_R1_001.fastq.gz | 5f6d41e67bb9cd595699f142772c19a5 | ftp://ftp-trace.ncbi.nih.gov/giab/ftp/data/NA12878/NIST_NA12878_HG001_HiSeq_300x/140407_D00360_0017_BH947YADXX/Project_RM8398/Sample_U5a/U5a_AGTCAA_L002_R2_001.fastq.gz | ab1459c355f2b9f10b49b69916c2c440 |
| ftp://ftp-trace.ncbi.nih.gov/giab/ftp/data/NA12878/NIST_NA12878_HG001_HiSeq_300x/140407_D00360_0017_BH947YADXX/Project_RM8398/Sample_U5a/U5a_AGTCAA_L002_R1_002.fastq.gz | efd34225c3fd8260dce7ec4f0a7fa6de | ftp://ftp-trace.ncbi.nih.gov/giab/ftp/data/NA12878/NIST_NA12878_HG001_HiSeq_300x/140407_D00360_0017_BH947YADXX/Project_RM8398/Sample_U5a/U5a_AGTCAA_L002_R2_002.fastq.gz | e34fd01cbf114f097dbe9bbbe771e950 |
| ftp://ftp-trace.ncbi.nih.gov/giab/ftp/data/NA12878/NIST_NA12878_HG001_HiSeq_300x/140407_D00360_0017_BH947YADXX/Project_RM8398/Sample_U5a/U5a_AGTCAA_L002_R1_003.fastq.gz | dc8537272d56330b72c4816ecded5b7d | ftp://ftp-trace.ncbi.nih.gov/giab/ftp/data/NA12878/NIST_NA12878_HG001_HiSeq_300x/140407_D00360_0017_BH947YADXX/Project_RM8398/Sample_U5a/U5a_AGTCAA_L002_R2_003.fastq.gz | 2176922f077e984a8a3558008e918c42 |
| ftp://ftp-trace.ncbi.nih.gov/giab/ftp/data/NA12878/NIST_NA12878_HG001_HiSeq_300x/140407_D00360_0017_BH947YADXX/Project_RM8398/Sample_U5a/U5a_AGTCAA_L002_R1_004.fastq.gz | 7a987c26fe210e4a3c3fbeaa7f46c967 | ftp://ftp-trace.ncbi.nih.gov/giab/ftp/data/NA12878/NIST_NA12878_HG001_HiSeq_300x/140407_D00360_0017_BH947YADXX/Project_RM8398/Sample_U5a/U5a_AGTCAA_L002_R2_004.fastq.gz | 397181f97691110c2feacbdc577ce7f7 |
| ftp://ftp-trace.ncbi.nih.gov/giab/ftp/data/NA12878/NIST_NA12878_HG001_HiSeq_300x/140407_D00360_0017_BH947YADXX/Project_RM8398/Sample_U5a/U5a_AGTCAA_L002_R1_005.fastq.gz | 68b59affcd377bc2c87c1a4ac91fa4f9 | ftp://ftp-trace.ncbi.nih.gov/giab/ftp/data/NA12878/NIST_NA12878_HG001_HiSeq_300x/140407_D00360_0017_BH947YADXX/Project_RM8398/Sample_U5a/U5a_AGTCAA_L002_R2_005.fastq.gz | 60a406888ae54bad012a99b600fa812b |
| ftp://ftp-trace.ncbi.nih.gov/giab/ftp/data/NA12878/NIST_NA12878_HG001_HiSeq_300x/140407_D00360_0017_BH947YADXX/Project_RM8398/Sample_U5a/U5a_AGTCAA_L002_R1_006.fastq.gz | 1bc4142f03872d50783041629f011257 | ftp://ftp-trace.ncbi.nih.gov/giab/ftp/data/NA12878/NIST_NA12878_HG001_HiSeq_300x/140407_D00360_0017_BH947YADXX/Project_RM8398/Sample_U5a/U5a_AGTCAA_L002_R2_006.fastq.gz | 86c7fbbb9958d0ece0e354b86b2acf53 |
| ftp://ftp-trace.ncbi.nih.gov/giab/ftp/data/NA12878/NIST_NA12878_HG001_HiSeq_300x/140407_D00360_0017_BH947YADXX/Project_RM8398/Sample_U5b/U5b_AGTTCC_L001_R1_001.fastq.gz | e953fc4b7ac6ad1e5a1a4b36b810983f | ftp://ftp-trace.ncbi.nih.gov/giab/ftp/data/NA12878/NIST_NA12878_HG001_HiSeq_300x/140407_D00360_0017_BH947YADXX/Project_RM8398/Sample_U5b/U5b_AGTTCC_L001_R2_001.fastq.gz | 4e283e91639a53c1ea681bfeb0a66e51 |
| ftp://ftp-trace.ncbi.nih.gov/giab/ftp/data/NA12878/NIST_NA12878_HG001_HiSeq_300x/140407_D00360_0017_BH947YADXX/Project_RM8398/Sample_U5b/U5b_AGTTCC_L001_R1_002.fastq.gz | 66ea82c7f934aaa1217be7f7cc09c6c5 | ftp://ftp-trace.ncbi.nih.gov/giab/ftp/data/NA12878/NIST_NA12878_HG001_HiSeq_300x/140407_D00360_0017_BH947YADXX/Project_RM8398/Sample_U5b/U5b_AGTTCC_L001_R2_002.fastq.gz | f2688c2942f552bcb3584e7a9b6b9260 |
| ftp://ftp-trace.ncbi.nih.gov/giab/ftp/data/NA12878/NIST_NA12878_HG001_HiSeq_300x/140407_D00360_0017_BH947YADXX/Project_RM8398/Sample_U5b/U5b_AGTTCC_L001_R1_003.fastq.gz | 864b1a4aa8e37ac053b8ef4b22b3dbc0 | ftp://ftp-trace.ncbi.nih.gov/giab/ftp/data/NA12878/NIST_NA12878_HG001_HiSeq_300x/140407_D00360_0017_BH947YADXX/Project_RM8398/Sample_U5b/U5b_AGTTCC_L001_R2_003.fastq.gz | 8d3d0c071e08f0d53670f3a26a391ecd |
| ftp://ftp-trace.ncbi.nih.gov/giab/ftp/data/NA12878/NIST_NA12878_HG001_HiSeq_300x/140407_D00360_0017_BH947YADXX/Project_RM8398/Sample_U5b/U5b_AGTTCC_L001_R1_004.fastq.gz | 03267925942876ccb7b608e7c0c9417a | ftp://ftp-trace.ncbi.nih.gov/giab/ftp/data/NA12878/NIST_NA12878_HG001_HiSeq_300x/140407_D00360_0017_BH947YADXX/Project_RM8398/Sample_U5b/U5b_AGTTCC_L001_R2_004.fastq.gz | b90111efec74b05b48286e67abb8451c |
| ftp://ftp-trace.ncbi.nih.gov/giab/ftp/data/NA12878/NIST_NA12878_HG001_HiSeq_300x/140407_D00360_0017_BH947YADXX/Project_RM8398/Sample_U5b/U5b_AGTTCC_L001_R1_005.fastq.gz | b84c7b6d6d901d292c7b1b8a7f810ec3 | ftp://ftp-trace.ncbi.nih.gov/giab/ftp/data/NA12878/NIST_NA12878_HG001_HiSeq_300x/140407_D00360_0017_BH947YADXX/Project_RM8398/Sample_U5b/U5b_AGTTCC_L001_R2_005.fastq.gz | 631c7a43fd796cbb4a6767387aff48c3 |
| ftp://ftp-trace.ncbi.nih.gov/giab/ftp/data/NA12878/NIST_NA12878_HG001_HiSeq_300x/140407_D00360_0017_BH947YADXX/Project_RM8398/Sample_U5b/U5b_AGTTCC_L002_R1_001.fastq.gz | 0c4d02aa3da6a9ec7deec7f3dd50b699 | ftp://ftp-trace.ncbi.nih.gov/giab/ftp/data/NA12878/NIST_NA12878_HG001_HiSeq_300x/140407_D00360_0017_BH947YADXX/Project_RM8398/Sample_U5b/U5b_AGTTCC_L002_R2_001.fastq.gz | 44c0b8a05c6b8dd1e1563efa3d59e14f |
| ftp://ftp-trace.ncbi.nih.gov/giab/ftp/data/NA12878/NIST_NA12878_HG001_HiSeq_300x/140407_D00360_0017_BH947YADXX/Project_RM8398/Sample_U5b/U5b_AGTTCC_L002_R1_002.fastq.gz | b1bd692a9e12cab5bc477f585ce33b95 | ftp://ftp-trace.ncbi.nih.gov/giab/ftp/data/NA12878/NIST_NA12878_HG001_HiSeq_300x/140407_D00360_0017_BH947YADXX/Project_RM8398/Sample_U5b/U5b_AGTTCC_L002_R2_002.fastq.gz | bac5bd25881e1e61418e56cfeeb43e2c |
| ftp://ftp-trace.ncbi.nih.gov/giab/ftp/data/NA12878/NIST_NA12878_HG001_HiSeq_300x/140407_D00360_0017_BH947YADXX/Project_RM8398/Sample_U5b/U5b_AGTTCC_L002_R1_003.fastq.gz | 583530483628695114bf3a71f1a54985 | ftp://ftp-trace.ncbi.nih.gov/giab/ftp/data/NA12878/NIST_NA12878_HG001_HiSeq_300x/140407_D00360_0017_BH947YADXX/Project_RM8398/Sample_U5b/U5b_AGTTCC_L002_R2_003.fastq.gz | 9683da893abac10bfc415ea3ac8f06e6 |
| ftp://ftp-trace.ncbi.nih.gov/giab/ftp/data/NA12878/NIST_NA12878_HG001_HiSeq_300x/140407_D00360_0017_BH947YADXX/Project_RM8398/Sample_U5b/U5b_AGTTCC_L002_R1_004.fastq.gz | a701a4da3c8cd21581bfc3751f2fad71 | ftp://ftp-trace.ncbi.nih.gov/giab/ftp/data/NA12878/NIST_NA12878_HG001_HiSeq_300x/140407_D00360_0017_BH947YADXX/Project_RM8398/Sample_U5b/U5b_AGTTCC_L002_R2_004.fastq.gz | fd7d71c8218895474ed143721be949aa |
| ftp://ftp-trace.ncbi.nih.gov/giab/ftp/data/NA12878/NIST_NA12878_HG001_HiSeq_300x/140407_D00360_0017_BH947YADXX/Project_RM8398/Sample_U5b/U5b_AGTTCC_L002_R1_005.fastq.gz | c27f3b45b11e85eb502acc731c39a635 | ftp://ftp-trace.ncbi.nih.gov/giab/ftp/data/NA12878/NIST_NA12878_HG001_HiSeq_300x/140407_D00360_0017_BH947YADXX/Project_RM8398/Sample_U5b/U5b_AGTTCC_L002_R2_005.fastq.gz | 088acb5be31346419008a31b25b9d2de |
| ftp://ftp-trace.ncbi.nih.gov/giab/ftp/data/NA12878/NIST_NA12878_HG001_HiSeq_300x/140407_D00360_0017_BH947YADXX/Project_RM8398/Sample_U5c/U5c_CCGTCC_L001_R1_001.fastq.gz | 52f3ed344d88278c703faa1280818ce1 | ftp://ftp-trace.ncbi.nih.gov/giab/ftp/data/NA12878/NIST_NA12878_HG001_HiSeq_300x/140407_D00360_0017_BH947YADXX/Project_RM8398/Sample_U5c/U5c_CCGTCC_L001_R2_001.fastq.gz | 8b3991a389c401c76d930803d0a4d334 |
| ftp://ftp-trace.ncbi.nih.gov/giab/ftp/data/NA12878/NIST_NA12878_HG001_HiSeq_300x/140407_D00360_0017_BH947YADXX/Project_RM8398/Sample_U5c/U5c_CCGTCC_L001_R1_002.fastq.gz | 2a54a6c21136b640e1e8da39c6b2183d | ftp://ftp-trace.ncbi.nih.gov/giab/ftp/data/NA12878/NIST_NA12878_HG001_HiSeq_300x/140407_D00360_0017_BH947YADXX/Project_RM8398/Sample_U5c/U5c_CCGTCC_L001_R2_002.fastq.gz | 78f6f7edc89f708ff5b94f8dc54e00b5 |
| ftp://ftp-trace.ncbi.nih.gov/giab/ftp/data/NA12878/NIST_NA12878_HG001_HiSeq_300x/140407_D00360_0017_BH947YADXX/Project_RM8398/Sample_U5c/U5c_CCGTCC_L001_R1_003.fastq.gz | 00db7ff0408319953d0bbb8e92e83867 | ftp://ftp-trace.ncbi.nih.gov/giab/ftp/data/NA12878/NIST_NA12878_HG001_HiSeq_300x/140407_D00360_0017_BH947YADXX/Project_RM8398/Sample_U5c/U5c_CCGTCC_L001_R2_003.fastq.gz | cbcb31d014e705508ecb569cd0d83521 |
| ftp://ftp-trace.ncbi.nih.gov/giab/ftp/data/NA12878/NIST_NA12878_HG001_HiSeq_300x/140407_D00360_0017_BH947YADXX/Project_RM8398/Sample_U5c/U5c_CCGTCC_L001_R1_004.fastq.gz | 40a22c0f14f10bfd73e33405cd747ba1 | ftp://ftp-trace.ncbi.nih.gov/giab/ftp/data/NA12878/NIST_NA12878_HG001_HiSeq_300x/140407_D00360_0017_BH947YADXX/Project_RM8398/Sample_U5c/U5c_CCGTCC_L001_R2_004.fastq.gz | 43b8abacb73c9a41a44c22d0d9f222f9 |
| ftp://ftp-trace.ncbi.nih.gov/giab/ftp/data/NA12878/NIST_NA12878_HG001_HiSeq_300x/140407_D00360_0017_BH947YADXX/Project_RM8398/Sample_U5c/U5c_CCGTCC_L001_R1_005.fastq.gz | f31757c2c1dc30af6ac908433bb93a47 | ftp://ftp-trace.ncbi.nih.gov/giab/ftp/data/NA12878/NIST_NA12878_HG001_HiSeq_300x/140407_D00360_0017_BH947YADXX/Project_RM8398/Sample_U5c/U5c_CCGTCC_L001_R2_005.fastq.gz | 9cc4a96cd8a3595066a3fb0a4d4870d5 |
| ftp://ftp-trace.ncbi.nih.gov/giab/ftp/data/NA12878/NIST_NA12878_HG001_HiSeq_300x/140407_D00360_0017_BH947YADXX/Project_RM8398/Sample_U5c/U5c_CCGTCC_L001_R1_006.fastq.gz | 2c120f3f37d60d9c673812ca33939d7f | ftp://ftp-trace.ncbi.nih.gov/giab/ftp/data/NA12878/NIST_NA12878_HG001_HiSeq_300x/140407_D00360_0017_BH947YADXX/Project_RM8398/Sample_U5c/U5c_CCGTCC_L001_R2_006.fastq.gz | 89ad37e9149865b936a6751af4079119 |
| ftp://ftp-trace.ncbi.nih.gov/giab/ftp/data/NA12878/NIST_NA12878_HG001_HiSeq_300x/140407_D00360_0017_BH947YADXX/Project_RM8398/Sample_U5c/U5c_CCGTCC_L002_R1_001.fastq.gz | afcd29ca24845ec6cbb8af37a7b6a0ca | ftp://ftp-trace.ncbi.nih.gov/giab/ftp/data/NA12878/NIST_NA12878_HG001_HiSeq_300x/140407_D00360_0017_BH947YADXX/Project_RM8398/Sample_U5c/U5c_CCGTCC_L002_R2_001.fastq.gz | eedc180944758a57721bb138d3d78b85 |
| ftp://ftp-trace.ncbi.nih.gov/giab/ftp/data/NA12878/NIST_NA12878_HG001_HiSeq_300x/140407_D00360_0017_BH947YADXX/Project_RM8398/Sample_U5c/U5c_CCGTCC_L002_R1_002.fastq.gz | 957be870479d54ba7f5e543ace8c82f9 | ftp://ftp-trace.ncbi.nih.gov/giab/ftp/data/NA12878/NIST_NA12878_HG001_HiSeq_300x/140407_D00360_0017_BH947YADXX/Project_RM8398/Sample_U5c/U5c_CCGTCC_L002_R2_002.fastq.gz | 464403671f22c79a313c034f586382b6 |
| ftp://ftp-trace.ncbi.nih.gov/giab/ftp/data/NA12878/NIST_NA12878_HG001_HiSeq_300x/140407_D00360_0017_BH947YADXX/Project_RM8398/Sample_U5c/U5c_CCGTCC_L002_R1_003.fastq.gz | 304723e410de4cb6a3f04540f48bb1cf | ftp://ftp-trace.ncbi.nih.gov/giab/ftp/data/NA12878/NIST_NA12878_HG001_HiSeq_300x/140407_D00360_0017_BH947YADXX/Project_RM8398/Sample_U5c/U5c_CCGTCC_L002_R2_003.fastq.gz | 67d84e1f917814371cba8f17efb0c600 |
| ftp://ftp-trace.ncbi.nih.gov/giab/ftp/data/NA12878/NIST_NA12878_HG001_HiSeq_300x/140407_D00360_0017_BH947YADXX/Project_RM8398/Sample_U5c/U5c_CCGTCC_L002_R1_004.fastq.gz | 7bf8566bada15b39c94d0041f6e36816 | ftp://ftp-trace.ncbi.nih.gov/giab/ftp/data/NA12878/NIST_NA12878_HG001_HiSeq_300x/140407_D00360_0017_BH947YADXX/Project_RM8398/Sample_U5c/U5c_CCGTCC_L002_R2_004.fastq.gz | ab5eb23fe534f3bc7b77c2bb33513e22 |
| ftp://ftp-trace.ncbi.nih.gov/giab/ftp/data/NA12878/NIST_NA12878_HG001_HiSeq_300x/140407_D00360_0017_BH947YADXX/Project_RM8398/Sample_U5c/U5c_CCGTCC_L002_R1_005.fastq.gz | 48e243a206da2e65a8fdaef20d883d01 | ftp://ftp-trace.ncbi.nih.gov/giab/ftp/data/NA12878/NIST_NA12878_HG001_HiSeq_300x/140407_D00360_0017_BH947YADXX/Project_RM8398/Sample_U5c/U5c_CCGTCC_L002_R2_005.fastq.gz | 7179328d092a9acbf762d2d2a841974f |
| ftp://ftp-trace.ncbi.nih.gov/giab/ftp/data/NA12878/NIST_NA12878_HG001_HiSeq_300x/140407_D00360_0017_BH947YADXX/Project_RM8398/Sample_U5c/U5c_CCGTCC_L002_R1_006.fastq.gz | fee4bd11c70763a46b3093be1bb11457 | ftp://ftp-trace.ncbi.nih.gov/giab/ftp/data/NA12878/NIST_NA12878_HG001_HiSeq_300x/140407_D00360_0017_BH947YADXX/Project_RM8398/Sample_U5c/U5c_CCGTCC_L002_R2_006.fastq.gz | b66027b226251e91f571c48ccf823273 |

We merged those files into a pair of FASTQ file as the order they are listed.

Here are the md5 number of the pair of files.

NA12878_1.fastq df992cf762aad70a8a8d1df447221f5e

NA12878_2.fastq 3f12fafce7e4b64b2e84a6a7d65c96bd

|  | Read | Base |
| --- | --- | --- |
| NA12878_1.fastq | 322939371 | 47795026908 |
| NA12878_2.fastq | 322939371 | 47795026908 |

Testing environment:

up to 16 nodes, each of which has 24 cores of Intel(R) Xeon(R) CPU E5-2620 v4 @ 2.10GHz and RAM of 128G. For SOAPnuke MapReduce, the data is stored in HDFS. For other usage, the data is stored in HDD.

Testing Tools:

Among the tools we surveyed on, we have chosen those workflow-like tools capable of adapter trimming, length filtering and quality filtering. Thus, 5 tools are selected for testing: SOAPnuke, Trimmomatic, BBDuk, AlienTrimmer, AfterQC. Functions and features of these tools can be accessed in Additional File 1.

Preprocessing settings:

Recognize adapter ‘AGATCGGAAGAGCACACGTCTGAACTCCAGTCA’ from read1 and trims from adapter to 3’ end

Recognize adapter ‘AGATCGGAAGAGCGTCGTGTAGGGAAAGAGTGT’ from read2 and trims from adapter to 3’ end

F ilter reads with average quality score less than 20 or with more than 50% bases with quality lower than 20.

Filter reads shorter than 15

Here are the parameters used in testing.

SOAPnuke:

/bin/time -f "%U(user) %S(system) %E(elapsed) %P(cpu) %M(max RAM KB)" SOAPnuke filter -1 NA12878_1.fastq -2 NA12878_2.fastq -l 0 -n 1 -m 20 -f AGATCGGAAGAGCACACGTCTGAACTCCAGTCA -r AGATCGGAAGAGCGTCGTGTAGGGAAAGAGTGT --thread 1 -Q 2 --sanger --minLen 15 -C nuke1.fq -D nuke2.fq

Trimmomatic:

/bin/time -f "%U(user) %S(system) %E(elapsed) %P(cpu) %M(max RAM KB)" java -jar trimmomatic-0.36.jar PE -threads 1 NA12878_1.fastq NA12878_2.fastq op1.fastq op2.fastq op3.fastq op4.fastq ILLUMINACLIP:TruSeqLT.fa:1:30:10 AVGQUAL:20 MINLEN:15

BBDuk:

/bin/time -f "%U(user) %S(system) %E(elapsed) %P(cpu) %Mcp (max RAM KB)" bbduk.sh in= NA12878_1.fastq in2= NA12878_2.fastq literal=AGATCGGAAGAGCACACGTCTGAACTCCAGTCA,AGATCGGAAGAGCGTCGTGTAGGGAAAGAGTGT out=bbres1.fastq out2=bbres2.fastq showspeed=f ktrim=r minlength=15 minavgquality=20 overwrite=t k=16

AlienTrimmer:

/bin/time -f "%U(user) %S(system) %E(elapsed) %P(cpu) %Mcp (max RAM KB)" java -jar AlienTrimmer.jar -if NA12878_1.fastq -ir NA12878_2.fastq -of alien1.fastq -or alien2.fastq -cf TruSeqLT1.fa -cr TruSeqLT2.fa -q 20 -p 75

AfterQC:

/bin/time -f "%U(user) %S(system) %E(elapsed) %P(cpu) %Mcp (max RAM KB)" pypy after.py -1 NA12878_1.fastq -2 NA12878_2.fastq -f 0 -t 0 -q 20 -u 75 -p 150 -n 150 -s 15

Adapter files format:

TruSeqLT.fa (used by Trimmomatic):

>Prefix/1

AGATCGGAAGAGCACACGTCTGAACTCCAGTCA

>Prefix/2

AGATCGGAAGAGCGTCGTGTAGGGAAAGAGTGT

TruSeqLT1.fa (used by AlienTrimmer):

AGATCGGAAGAGCACACGTCTGAACTCCAGTCA

TruSeqLT2.fa (used by AlienTrimmer):

AGATCGGAAGAGCGTCGTGTAGGGAAAGAGTGT

Benchmark result:

Performance:

|  | Time(min) | Speed (read/s) | CPU | Max RAM（GB） |
| --- | --- | --- | --- | --- |
| SOAPnuke (1 node 1 thread) | 302.7 | 33947.8 | 250% | 0.62 |
| SOAPnuke (16 nodes) | 9.4 | 1093191.1 | 640% | 50.10 |
| Trimmomatic (1 thread) | 84.7 | 121380.1 | 75% | 2.98 |
| Trimmomatic (24 threads) | 50.5 | 203582.1 | 239% | 10.28 |
| BBDuk | 57.2 | 162230.2 | 259% | 11.40 |
| AlienTrimmer | 530.2 | 19076.1 | 99% | 0.54 |
| AfterQC (pypy) | 2482.7 | 4319.1 | 99% | 0.21 |

From the table we know that, AfterQC is the tool occupying least resources, but its processing time is too long for practical usage, especially considering we ran the program with pypy, which is the faster method of running. Among the remaining tools, SOAPnuke is good at keeping balance between resources occupancy and performance. If high performance is demanded, users can choose to run SOAPnuke on multiple nodes with MapReduce framework. In our testing, 16 nodes can achieve ~32 times acceleration, which is 5.37 times faster than the best speed of other tools.

To evaluate the effect of preprocessing, we performed downstream analyses of these preprocessed data. GATK best practice (<https://software.broadinstitute.org/gatk/best-practices/)> is applied in the pipeline. Data is processed by Alignment, rmdup, baseRecal, bamSort and haplotypeCaller modules in order. The default parameters are set for testing.

For the alignment, hg38 is used as reference.

For the haplotypeCaller, GIAB NIST v3.3.2 (<ftp://ftp-trace.ncbi.nlm.nih.gov/giab/ftp/release/NA12878_HG001/NISTv3.3.2/)> is used for comparison. Here is the result.

|  | Bases_trim | read_Filter_Q | read_Filter_L | Left read | Left bases |
| --- | --- | --- | --- | --- | --- |
| SOAPnuke | / | 6740528 | 23217750 | 308279886*2 | 91,250,846,256 |
| Trimmomatic | / | / | / | 308426916*2 | 91,294,367,136 |
| BBDuk | 49836723 | 89048354 | 56450 | 278386969*2 | 82,365,013,737 |
| AlienTrimmer | / | / | / | 303423939*2 | 88,814,612,909 |
| AfterQC | 210283778 | 890667 | 358852 | 321689852*2 | 95,013,502,252 |

|  | Mapping Rate | SNPs Precision | SNPs Sensitivity | SNPs F-measure | INDELs Precision | INDELs Sensitivity | INDELs F-measure |
| --- | --- | --- | --- | --- | --- | --- | --- |
| SOAPnuke | 99.72% | 0.9967 | 0.9811 | 0.9888 | 0.9806 | 0.9575 | 0.9689 |
| Trimmomatic | 99.71% | 0.9966 | 0.9811 | 0.9888 | 0.9806 | 0.9575 | 0.9689 |
| BBDuk | 99.69% | 0.9966 | 0.9797 | 0.9881 | 0.9698 | 0.9184 | 0.9434 |
| AlienTrimmer | 99.67% | 0.9954 | 0.9810 | 0.9882 | 0.9792 | 0.9540 | 0.9665 |
| AfterQC | 99.41% | 0.9968 | 0.9811 | 0.9889 | 0.9811 | 0.9586 | 0.9697 |

And here is the result of alignment and variant calling:

Mapping rate is the ratio between mapped reads and all reads, which reflects the impact of preprocessing on alignment result. F-measure is a measure of a test's accuracy, which considers both the precision and recall of the test. SNPs and indels are two main categories of variants, and F-measure is computed for them respectively. In short, higher F-measure indicates better preprocessing effect.
