# Supplementary figures and images for: SOAPnuke: a MapReduce acceleration-supported software for integrated quality control and preprocessing of high-throughput sequencing data
Source: Gigascience. 2017 Dec 4;7(1):gix120. doi: 10.1093/gigascience/gix120 (PMC5788068; doi:10.1093/gigascience/gix120)

Quality Control

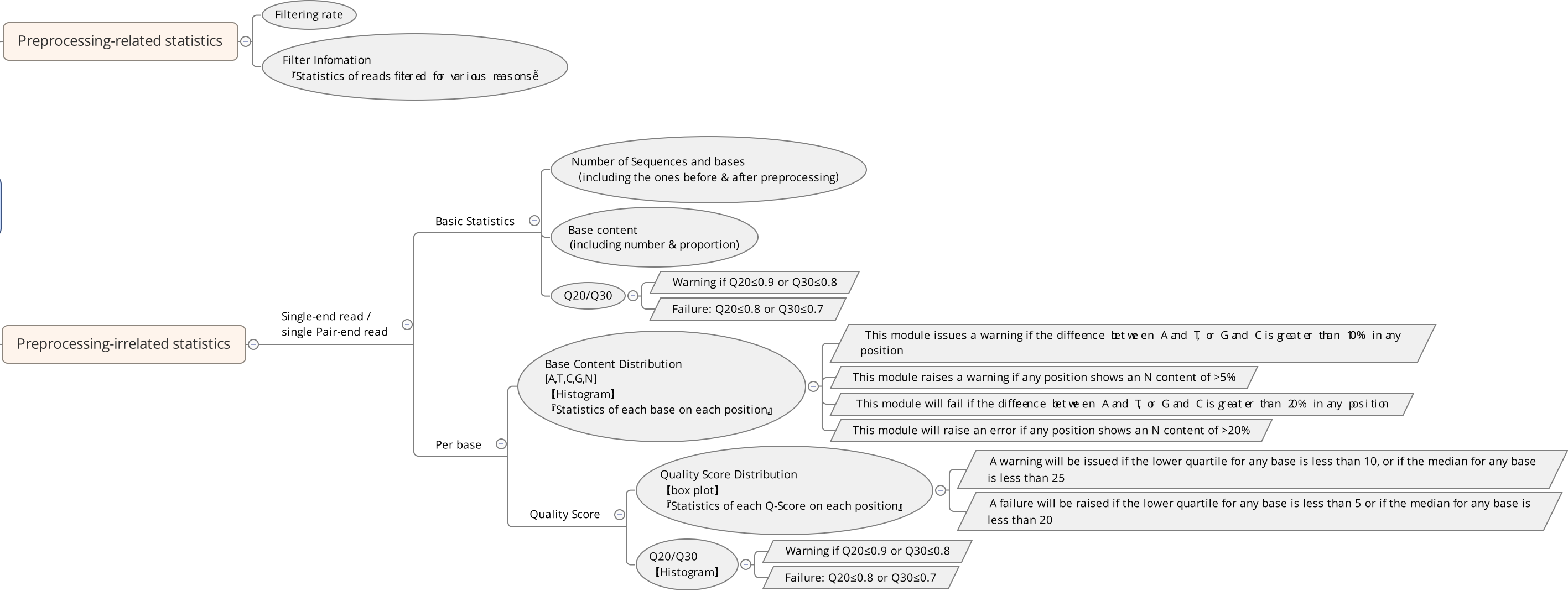

Supplement: Supplement materials [file gix120_supp.zip › SM2.pdf]
