# Supplementary material for: SOAPnuke: a MapReduce acceleration-supported software for integrated quality control and preprocessing of high-throughput sequencing data
Source: Gigascience. 2017 Dec 4;7(1):gix120. doi: 10.1093/gigascience/gix120 (PMC5788068; doi:10.1093/gigascience/gix120)
Supplement: Response_to_Reviewer_Comments_Original_Submission.pdf [file gix120_response_to_reviewer_comments_original_submission.pdf]

Author's Response To Reviewer Comments

Dear Reviewer:

Thank you for your letter. We were pleased to know that our manuscript was rated as potentially acceptable for publication in GigaScience, subject to adequate revision and response to your comments.

We revised the manuscript following the instructions provided in your letter. To address the first question, two sentences has been added to the DISCUSSION section: ‘To users without computing cluster, SOAPnuke might not be an optimal tool in terms of overall performance. Thus, we are performing refactoring to increase the standalone processing speed’. As suggested in the second question, Variant calling results and corresponding description have been added to RESULT section. We also greatly appreciate the suggestions offered in third question and have changed the wording and bottom-half content. In terms of language editing, we have fixed the problems you indicated and re-collated the whole manuscript.

We would like to express our sincere gratitude to you for improving the quality of our manuscript with helpful suggestions.

Thank you.

Best Regards,
Magic Fang
