# Supplementary material for: SOAPnuke: a MapReduce acceleration-supported software for integrated quality control and preprocessing of high-throughput sequencing data
Source: Gigascience. 2017 Dec 4;7(1):gix120. doi: 10.1093/gigascience/gix120 (PMC5788068; doi:10.1093/gigascience/gix120)
Supplement: Reviewer_1_Report_(Original_Submission) -- Joshua W. K. Ho — 03 Aug 2017 Reviewed [file gix120_reviewer_1_report_(original_submission).pdf]

Reviewer Report

Title: SOAPnuke: A MapReduce Acceleration supported Software for integrated Quality Control and Preprocessing of High-Throughput Sequencing Data

Version: Original Submission **Date:** 8/3/2017

Reviewer name: Joshua W. K. Ho

Reviewer Comments to Author:

This revised version of the manuscript is an improvement over the previous version. I still have a few questions:1. In Table 1, it is clear that the faster performance of SOAPnuke comes with a significant highly cost in terms of CPU and memory usage. This translates to higher cost (in terms of using on-demand cloud computing infrastructure or access to a private compute cluster). I note that if only 1 thread was used, trimmomatic is substantially faster than SOAPnuke. Therefore it appears to me that the real benefit of SOAPnuke is for users who have access to a large compute cluster in order to make full use of the MapReduce capability.2. The table about comparison of variant calling results in Additional File 4 is essential for interpretation of the results. I suggest putting it in the main figure and properly explain and discuss the results.3. Discussion, starting on line 205: 'Secondly, the QC statistics are of limited help to judge the availability of data…' I think this statement is quite strange given the premise of this paper is that QC is important. The example given after this statement is problematic - surely the total number of mappable reads (which is an important quality measure) will drop while increasing the threshold for filtering low-quality reads. Therefore the statement that suggest 'all' quality-related QC stats will persist rising (note incorrect grammar here)…is not accurate. I would have thought that a reasonable user should be able to determine the quality of the data using a comprehensive list of QC statistics.4. I still think this manuscript requires further language editing. Here are a few examples:line 28: 'an abundant-function tool with 'QC-PreprocessQC' architecture' => 'a tool that provide many functions for a 'QC-Preprcess-QC' workflow'line 198: 'Datasets from the same….are tend to' => 'Datasets from the same experiments….tend to'line 207: 'QC stats' => 'QC statistics' (please note that there are many occurrence of this shorthand. 'stats' is not a proper English word. Please check the whole manuscript.

Level of Interest

Please indicate how interesting you found the manuscript: An article whose findings are important to those with closely related research interests

Quality of Written English

Please indicate the quality of language in the manuscript: Needs some language corrections before being published

Declaration of Competing Interests

Please complete a declaration of competing interests, considering the following questions:

- Have you in the past five years received reimbursements, fees, funding, or salary from an organisation that may in any way gain or lose financially from the publication of this manuscript, either now or in the future?
- Do you hold any stocks or shares in an organisation that may in any way gain or lose financially from the publication of this manuscript, either now or in the future?
- Do you hold or are you currently applying for any patents relating to the content of the manuscript?
- Have you received reimbursements, fees, funding, or salary from an organization that holds or has applied for patents relating to the content of the manuscript?
- Do you have any other financial competing interests?
- Do you have any non-financial competing interests in relation to this paper?

If you can answer no to all of the above, write 'I declare that I have no competing interests' below. If your reply is yes to any, please give details below.

I declare that I have no competing interests.

I agree to the open peer review policy of the journal. I understand that my name will be included on my report to the authors and, if the manuscript is accepted for publication, my named report including any attachments I upload will be posted on the website along with the authors' responses. I agree for my report to be made available under an Open Access Creative Commons CC-BY license (http://creativecommons.org/licenses/by/4.0/). I understand that any comments which I do not wish to be included in my named report can be included as confidential comments to the editors, which will not be published.

I agree to the open peer review policy of the journal
